# Analyses of oligodontia phenotypes and genetic etiologies

**DOI:** 10.1038/s41368-021-00135-3

**Published:** 2021-09-30

**Authors:** Mengqi Zhou, Hong Zhang, Heather Camhi, Figen Seymen, Mine Koruyucu, Yelda Kasimoglu, Jung-Wook Kim, Hera Kim-Berman, Ninna M. R. Yuson, Paul J. Benke, Yiqun Wu, Feng Wang, Yaqin Zhu, James P. Simmer, Jan C-C. Hu

**Affiliations:** 1 Dental Research Laboratory, University of Michigan School of Dentistry, Ann Arbor, MI USA; 2 Department of Second Dental Center, Shanghai Ninth People’s Hospital, Shanghai Jiao Tong University School of Medicine; College of Stomatology, Shanghai Jiao Tong University; National Center for Stomatology; National Clinical Research Center for Oral Diseases; Shanghai Key Laboratory of Stomatology, Shanghai, China; 3 Department of General Dentistry, Shanghai Ninth People’s Hospital, Shanghai Jiao Tong University School of Medicine; College of Stomatology, Shanghai Jiao Tong University; National Center for Stomatology; National Clinical Research Center for Oral Diseases; Shanghai Key Laboratory of Stomatology, Shanghai, China; 4 Orthodontic and Pediatric Dentistry, University of Michigan School of Dentistry, 1011N. University Ave, Ann Arbor, MI USA; 5 Mott Children’s Health Center 806 Tuuri Place, Flint, MI USA; 6 Department of Pedodontics, Faculty of Dentistry, Istanbul University, Istanbul, Turkey; 7 Department of Molecular Genetics & Dental Research Institute School of Dentistry, Seoul National University, Seoul, Korea; 8 Department of Pediatric Dentistry & Dental Research Institute School of Dentistry, Seoul National University, Seoul, Korea; 9 Department of Paediatric Dentistry, Women’s and Children’s Hospital, North Adelaide, SA Australia; 10 Department of Medical Genetics, Joe DiMaggio Children’s Hospital, Hollywood, FL USA; 11 Department of Oral Implantology, Shanghai Ninth People’s Hospital, Shanghai Jiao Tong University School of Medicine; College of Stomatology, Shanghai Jiao Tong University; National Center for Stomatology; National Clinical Research Center for Oral Diseases; Shanghai Key Laboratory of Stomatology, Shanghai, China

**Keywords:** Dental diseases, Disease genetics

## Abstract

Oligodontia is the congenital absence of six or more teeth and comprises the more severe forms of tooth agenesis. Many genes have been implicated in the etiology of tooth agenesis, which is highly variable in its clinical presentation. The purpose of this study was to identify associations between genetic mutations and clinical features of oligodontia patients. An online systematic search of papers published from January 1992 to June 2021 identified 381 oligodontia cases meeting the eligibility criteria of causative gene mutation, phenotype description, and radiographic records. Additionally, ten families with oligodontia were recruited and their genetic etiologies were determined by whole-exome sequence analyses. We identified a novel mutation in *WNT10A* (c.99_105dup) and eight previously reported mutations in *WNT10A* (c.433 G > A; c.682 T > A; c.318 C > G; c.511.C > T; c.321 C > A), *EDAR* (c.581 C > T), and *LRP6* (c.1003 C > T, c.2747 G > T). Collectively, 20 different causative genes were implicated among those 393 cases with oligodontia. For each causative gene, the mean number of missing teeth per case and the frequency of teeth missing at each position were calculated. Genotype–phenotype correlation analysis indicated that molars agenesis is more likely linked to *PAX9* mutations, mandibular first premolar agenesis is least associated with *PAX9* mutations. Mandibular incisors and maxillary lateral incisor agenesis are most closely linked to *EDA* mutations.

## Introduction

Congenital absence of teeth or hypodontia is the most commonly encountered dominant human disorder, while oligodontia is a less common dominant disorder with a hallmark presentation of congenital absence of six or more teeth, excluding third molars.^1,2^ Oligodontia can present as an isolated trait such as in tooth agenesis or exist as part of a syndrome.^3^ When oligodontia is part of a syndrome, concomitant abnormalities may be observed in the skin, nails, eyes, ears, and skeleton.^4^

The prevalence of oligodontia varies based on reports from different geographic regions and ethnicities. The prevalence of oligodontia was estimated as 0.19% in a Swedish study,^5^ 0.08% in a Dutch study,^1^ and 0.1% in a Finnish study.^6^ The prevalence of oligodontia in the Caucasian populations in North America, Australia, and Europe is estimated to be 0.14%,^7^ and ~0.25% in Asian populations in China.^8^ However, articles reporting oligodontia prevalence were extremely limited for Africa. The prevalence also varies among different age groups. According to studies from northern Europe, oligodontia occurs in 0.16% of the school children at 9–13 years of age,^9^ 0.084% among 18-year-old young adults.^10^ Although it was reported that the prevalence of tooth agenesis in females was higher than in males,^11^ no additional literature has offered a logical explanation.

In addition to genetic factors, studies have suggested that tooth agenesis can also be influenced by many environmental and/or host factors, such as radiotherapy, chemotherapy, disease/infection of the preceding deciduous teeth,^12^ viral infection during pregnancy, and metabolic imbalances, etc.^13^. In this study, we focus only on oligodontia caused by genetic factors. Third molars were excluded from this analysis, which are often ignored in oligodontia studies because of the high frequency of their absence (~20%) in the general population, although third molar absence is significantly higher in persons missing teeth other than third molars.^14^ Furthermore, ten families with oligodontia were recruited and characterized with clinical examinations and the causative mutation determined by genotype analyses.

The fact that oligodontia can be presented as an isolated condition or as part of a syndrome makes accurate diagnosis important for early detection and management. In many clinical settings, where a genetic diagnosis is not possible, determining the potential correlation between the oligodontia genotype and phenotype may provide useful insights leading to early and accurate clinical diagnoses. The objective of this study was to characterize and correlate oligodontia phenotypes with their causative genetic etiologies, based upon analyses of available cases through a systematic review (Fig. S1) supplemented with a characterization of ten additional families with oligodontia.

## Results

### Mutational analysis and phenotypic findings

Ten probands with oligodontia were identified by groups working in North America and Asia. All of the probands had agenesis of six or more teeth (excluding third molars), and normal facial features, hair, skin, sweat glands, and nails determined through clinical examinations. In these families, we identified six different oligodontia-causing sequence variants (one novel) in seven unrelated *WNT10A* kindreds, two oligodontia-causing variants in *LRP6*, and one in *EDAR*.

**Fig. 1:**
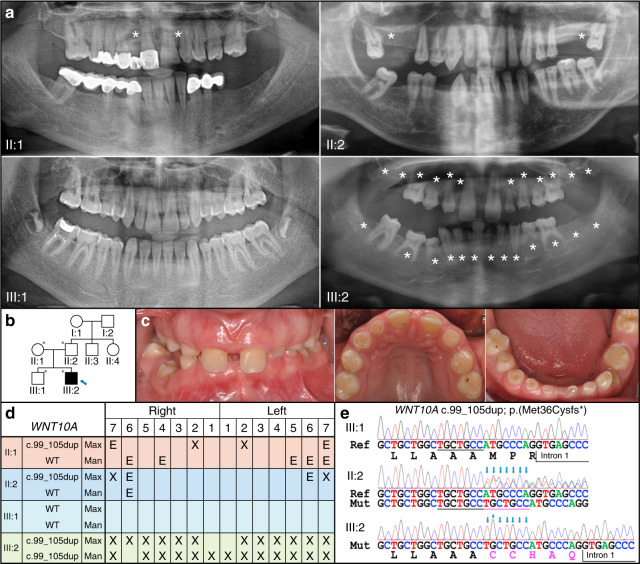
Oligodontia Family 1 from Turkey with the homozygous *WNT10A* exon 1 defect c.99_105dup, p.Met36CysfsTer10. **a** Panoramic radiographs with sites of tooth agenesis marked by stars. The proband (III:2) was the youngest at 13 years 4 months. **b** Pedigree of Family 1. Asterisks mark the four subjects who participated in the study. Subject genotypes are II:1 +/−, II:2 +/−, III:1 +/+, and III:2 −/−. **c** Oral photographs of the proband (III:2) showing marked attrition of retained primary teeth. **d** Summary chart of missing teeth (X, agenesis; E, extracted). The proband exhibited agenesis of 25 permanent teeth and was included in the oligodontia data analysis. **e** *WNT10A* chromatogram showing the wild-type (reference) sequence (III:1), heterozygous (II:2), and homozygous (III:2) conditions. The seven duplicated nucleotides (TGCTGCC) are indicated by blue arrows. Note the consistent dose effect of the mutation. The heterozygous parents both exhibited agenesis of two permanent teeth and the homozygous proband 25. The NCBI reference sequence designation for this *WNT10A* variant is NG_012179.1: g.5562_5568dup; NG_012179.1(WNT10A_v001): c.99_105dup, p.(Met36Cysfs*10)

Family 1 was a Turkish family reported to have no consanguinity (Fig. 1). The parents of the proband both exhibited agenesis of two permanent teeth and were each heterozygous for novel *WNT10A* 7-nucleotide duplication in exon 1. The NCBI reference sequence designation for this *WNT10A* variant is NG_012179.1: g.5562_5568dup; NG_012179.1(WNT10A_v001): c.99_105dup, p.(Met36Cysfs*10). The father’s tooth agenesis was two maxillary lateral incisors while the mother’s was two maxillary second molars. The proband who was homozygous for the WNT10 variant developed only three permanent teeth, missing 25 permanent teeth excluding third molars (which were also absent). At age 13 years 6 months 17 of his primary teeth were retained but had undergone significant attrition. His maxillary central incisors showed a narrower mesiodistal width and a lack of mesial and distal contour. His only permanent molar (#30) showed a rounded crown with decreased crown height. His older brother showed the wild-type sequence on both alleles and had no teeth missing. In this family, the *WNT10A* defect demonstrated a consistent dose effect.

In the supplemental data, we present six more families with oligodontia caused by *WNT10A* defects that have been previously described. In the first three, there is a similar dose effect as in Family 1. In Family 2 from Turkey (Fig. S2), serious oligodontia (agenesis of 22 and 24 teeth) was observed in the two compound heterozygotes for *WNT10A* defects c.433 G > A/p.Val145Met and c.682 T > A/p.Phe228Ile, whereas only four teeth failed to develop in the c.433 G > A or c.682 T > A heterozygotes. In Family 3 from Turkey (Fig. S3), the proband was homozygous for the *WNT10A* defect c.433 G > A/p.Val145Met and exhibited agenesis of 18 permanent teeth, whereas the heterozygous mother developed all of her permanent teeth and the father all but one (tooth #26). In Family 4 (Fig. S4), agenesis of 18 teeth was observed in the proband who was a compound heterozygote with the *WNT10A* defects c.318 C > G, p.Asn106Lys and c.682 T > A, p.(Phe228Ile), whereas her mother, who was heterozygous for only the c.318 C > G variation, developed all of her teeth. In Family 5 (Fig. S5) there were three subjects that were heterozygous for the *WNT10A* defect c.682 T > A, p.(Phe228Ile). One of the heterozygotes developed all of their teeth. The others showed agenesis of five and nine permanent teeth. In Family 6 (Fig. S6) there were four subjects heterozygous for the *WNT10A* defect c.321 C > A, p.Cys107*. Two showed oligodontia, failing to develop 6 and 14 permanent teeth. The number of missing teeth could not be ascertained for the other two. In Family 7 (Fig. S7) there were two heterozygous and three homozygous subjects with the *WNT10A* defect: c.682 T > A, p.Phe228Ile. The two heterozygotes failed to form zero and one permanent tooth. Two of the homozygotes weren’t much different, with only one and two permanent teeth failing to develop. The third homozygote, however, exhibited agenesis of 15 permanent teeth. Subjects II:1 and III:2 also carried a heterozygous *EDARADD* variation NM_145861.4: c.308 C > T; p.(Ser103Phe) (rs114632254), which is described in the literature as a functional variant.^15^

In Family 8 the proband showed agenesis of 15 permanent teeth, taurodontism, and microdontia caused by the heterozygous *EDAR* defect c.581 C > T, p.(Thr194Ile) (Fig. S8). In Family 9 the proband showed agenesis of 15 permanent teeth and taurodontism caused by the heterozygous *LRP6* defect c.1003 C > T, p.(Arg335*) (Fig. S9). In Family 10 the proband showed agenesis of 12 permanent teeth caused by the heterozygous *LRP6* defect c.2747 G > T, p.(Cys916Phe) (Fig. S10). A summary of mutations and corresponding phenotypes is provided in Table 1.

**Table 1:** Summary of mutations and phenotypes in *WNT10A, EDAR*, and *LRP6* genes

Family	Patient	Gender	Gene	Zygosity	Reference/Mutation	Teeth Missing	Phenotype	SIFT	PolyPhen-2	GnomAD exome frequency	dbSNP (154)
1	III:2	M	*WNT10A*	Homozygous	NG_012179.1:g.5522_5568dup NM_025216.2:c.99_105dup NP_079492.2:p.(Met36Cysfs*)	25	Hypoplastic enamel Ankylosis; Microdontia	N/A	N/A	N/A	N/A
2	V:2	F	*WNT10A*	Compound Heterozygous	NG_012179.1:g.14508G>A NM_025216.2:c.433G>A NP_079492.2:p.(Val145Met)	20	No disto-palatal cusp on maxillary first molars; conically shaped incisors; Over-retained 1˚ teeth.	0 0.002	1 0.999	N/A 0.013 916	rs543063101 rs121908120
2	V:4	M	*WNT10A*	Compound Heterozygous	NG_012179.1:g.14757T>A NM_025216.2:c.682T>A NP_079492.2: p.(Phe228Ile)	24		0 0.002	1 0.999	N/A	rs543063101 rs121908120
3	IV:1	F	*WNT10A*	Homozygous	NG_012179.1:g.14508G>A NM_025216.2:c.433G>A NP_079492.2:p.(Val145Met)	18	Attrition on 1˚ molars; Conically shaped incisors; Prominent mesio-palatal cusps on maxillary molars.	0	1	N/A	rs543063101
4	III:3	F	*WNT10A*	Compound Heterozygous	NG_012179.1: g.6833C>G NM_025216.2:c.318C>G NP_079492.2:p.(Asn106Lys) NG_012179.1:g.14757T>A NM_025216.2:c.682T>A NP_079492.2:p.(Phe228Ile)	19	Conically shaped 1˚ canine; enamel hypoplasia; moderate attrition; no disto-palatal cusps on max first molars; rounded crown morphology on mandibular first molars.	0.002	1	N/A	N/A
5	II:2	F	*WNT10A*	Heterozygous	NG_012179.1:g.14757T>A NM_025216.2:c.682T>A NP_079492.2:p.(Phe228Ile)	9	Microdontia; over-retained primary teeth.	0.002	0.989	0.013 916	rs121908120
6	II:5	M	*WNT10A*	Heterozygous	NG_012179.1: g.6836C>A NM_025216.2:c.321C>A NP_079492.2:p.(Cys107Ter)	6	Taurodontism	N/A	N/A	0.000 621	rs121908119
	III:2	F	*WNT10A*	Heterozygous	NG_012179.1: g.6836C>A NM_025216.2:c.321C>A NP_079492.2:p.(Cys107Ter)	14					
7	III:2	M	*WNT10A*	Homozygous	NG_012179.1:g.14757T>A NM_025216.2: c.682T>A NP_079492.2: p.(Phe228Ile)	15	Taurodontism; fusion of maxillary molar roots; Retained primary teeth.	0.002	0.999	0.013 916	rs121908120
8	III:4	F	*EDAR*	Heterozygous	NG_008257.1:g.83352C>T NM_022336.3:c.581C>T NP_071731.1:p.(Thr194Ile)	15	Taurodontism; Microdontia	0.012	0.999	0.000 004	rs143058412
9	III:3	M	*LRP6*	Heterozygous	NM_002336.2:c.1003C>T NP_002327.2:p.(Arg335*)	16		N/A	N/A	N/A	rs1565611848
10	III:1	M	LRP6	Heterozygous	NM_002336.2:c.2747G>T NP_002327.2:p.(Cys916Phe)	12		0	1	N/A	N/A

### Literature search results

Taken together, the data presented in 381 published cases and 12 cases from those ten study families were compiled and analyzed. The frequency of tooth absence at each tooth position was different among various causative genes. Twelve genes (frequency of mutations) including *PAX9* (24.7%)*, MSX1* (14.5%)*, WNT10A* (26.0%)*, WNT10B* (1.5%)*, AXIN2* (6.1%)*, EDA* (5.9%)*, EDAR* (4.0%)*, EDARADD* (1.3%)*, LRP6* (4.1%), *KREMEN1* (3.8%), *PITX2* (2.8%), and *SMOC2* (1.5%) that collectively accounted for 99.1% of cases were specifically analyzed in the following section.

### Data analyses

After combining data from our collected cases with data from published cases, the frequencies of missing teeth at each location were summarized in Table 2. It is demonstrated that the specific genetic etiology impacts the frequencies of tooth absence at each tooth position.

**Table 2:** Frequencies of tooth absence (in percentage) among the study cases affected by *PAX9, MSX1, WNT10A, WNT10B, AXIN2, EDA, EDAR, EDARADD, LRP6, KREMEN1, SMOC2*, and *PITX2* gene mutations

Please note that the same type of tooth absent in the right and left arches were pooled together for the calculation. Numbers in gold color represent the highest frequency and numbers in blue color represent the lowest frequency of tooth type absence in each causative gene

*Ca* canine; *CI* central incisor; *LI* lateral incisor; *Mo* molar; *PM* premolar

Seventy-four isolated oligodontia cases and twenty-eight syndromic oligodontia cases with *WNT10A* gene mutations (102 total) were analyzed. In our study, 53.84% of syndromic oligodontia cases were caused by *WNT10A* mutations. On average, (13.0 ± 6.2) (mean ± SD) teeth were missed per case. What stands out is the high frequency of absent maxillary second premolars (max. PM2, 82.4%), and the low frequency of absent maxillary central incisors (max. CI, 6.4%) (Table 2). WNT10B is most homologous to WNT10A (59.2% amino acid sequence identity).^16^ However, the phenotypic features associated with *WNT10A* and WNT10B mutations were different. The most frequently missing permanent teeth in persons with *WNT10B* mutations were mandibular lateral incisors (mand. LI, 91.7%) and the least were mandibular first premolars (8.3%). The average number of teeth missing per case was 13.7 ± 6.2.

*PAX9* is the most frequently involved gene responsible for isolated oligodontia.^17^ In this study, there were no cases diagnosed as syndromic oligodontia reported with *PAX9* mutations. On average, (11.7 ± 3.8) teeth were missed per case with *PAX9* mutations. A high percentage of agenesis was observed for maxillary second molars (max. Mo2, 86.6%), mandibular second molars (man. Mo2, 86.6%), maxillary second premolars (max. PM2 70.6%), and maxillary first molars (max. Mo1, 69.1%) (Table 2). In contrast, only 6.7% of maxillary central incisors were missing. This finding is consistent with a previous study.^18^ Interestingly, according to the published reports, cases with *PAX9* mutations showed a reduction in bitter taste perception.^19^

Fifty-seven oligodontia cases with *MSX1* mutations were analyzed. On average, (11.1 ± 3.8) teeth were absent per case when *MSX1* was mutated. Agenesis affected mainly premolars: mand. PM2 (88.6%), max. PM2 (84.2%), and max. PM1 (75.4%) (Table 2).

Twenty-four oligodontia cases with *AXIN2* gene mutations were evaluated. On average, (13.0 ± 5.6) teeth were missed per case. The most prevalent missing teeth were max. PM2 (83.3%) followed by mand. PM2 (83.3%). Less common agenesis observed was mandibular canine (mand. CA, 18.8%), max. Mo1 (33.3%), mand. Mo1 (37.5%), and maxillary central incisor (max. CI, 8.3%) (Table 2).

Twenty-three oligodontia cases with *EDA* mutations were analyzed. On average, (12.6 ± 5.6) teeth were missed per case. A high percentage of agenesis of mand. LI (91.3%), mand. CI (89.1%), and max. LI (84.8%) was noted (Table 2). Fifteen cases were reported to have *EDAR* gene mutations. On average, (10.5 ± 4.7) teeth were missed per case. The highest percentage of missing teeth were max. LI (66.7%), and mand. LI (66.7%). All cases with *EDAR* gene mutations showed no missing maxillary central incisors. Five cases diagnosed as isolated oligodontia with the *EDARADD* gene mutation were assessed. On average, (7.8 ± 2.2) teeth were missing per case. The tooth agenesis pattern was unique: 100% absence of the mandibular second premolars, but no missing central incisors, maxillary molars, mandibular first molars, mandibular canines, and mandibular lateral incisors.

Sixteen oligodontia cases with *LRP6* gene mutations were reviewed. On average, (13.8 ± 3.8) teeth were missing per case. The highest rate of agenesis was the max. LI (93.8%) and the lowest was max. CI (0.0%) (Table 2). Fifteen oligodontia cases with *KREMEN1* mutations were reviewed. On average, (10.1 ± 4.9) teeth were missing per case. The most prevalent missing teeth were max. LI (80.0%), mand. LI (80.0%), and mand. CI (80.0%) (Table 2). Eleven cases with *PITX2* mutations were evaluated. (16.8 ± 4.9) teeth were missing per case on average. The most prevalent missing teeth was Max. CI (90.9%), and the least prevalent missing teeth was Man. Mo1 (0.0%). Six cases with *SMOC2* mutations were evaluated. On average, (10.3 ± 2.6) teeth were missing per case. The most prevalent missing tooth types were max. PM2 (100.00%), and mand. PM2 (100.00%) (Table 2).

**Fig. 2:**
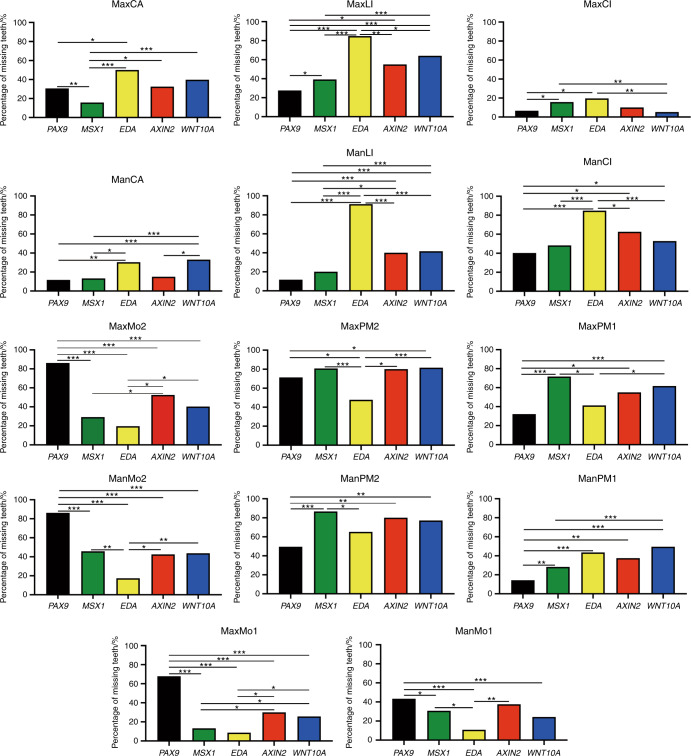
Correlations between the percentage of missing teeth at each tooth position (pooling right and left sides) and the causative gene *PAX9, MSX1, EDA, AXIN2*, and *WNT10A*. Max, maxillary; Mand, mandibular; Mo2, second molar; Mo1, first molar; PM2, second premolar; PM1, first premolar; Ca, canine; LI, lateral incisor; CI, central incisor. *P* value is marked with *<0.05, **<0.01, and ***<0.001

**Fig. 3:**
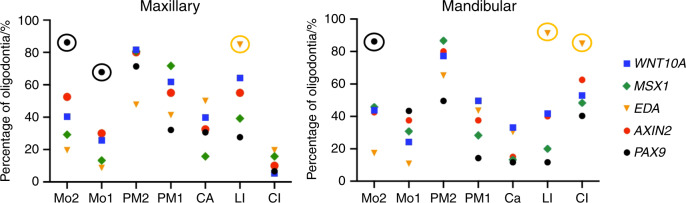
Correlations between genotype and dental phenotype in oligodontia cases. Each point represents the percentage of oligodontia cases caused by defects in a particular gene that fail to develop the type of tooth listed beneath the point. When a defective gene shows a significantly higher percentage of agenesis than other genes for a particular type of tooth, the point is circled. For instance when *PAX9* is defective, maxillary first and second molars and mandibular second molars have a significantly higher percentage of agenesis than when other genes are mutated. Similarly, when EDA is mutated the maxillary lateral and mandibular central and lateral incisors are more frequently absent than when other genes are mutated. Ca, canine; CI, central incisor; LI, lateral incisor; Mo, molar; PM, premolar

Considering the quantity of cases, we chose the top five genes (*PAX9*, *MSX1*, *EDA*, *AXIN2*, and *WNT10A*) for statistical analysis. The correlation between genotype and dental phenotype of oligodontia is shown in Figs. 2 and 3. At the maxillary second molar, maxillary first molar, mandibular first molar, and mandibular second molar positions, *PAX9* mutations showed a significantly higher percentage of missing teeth (*P* < 0.05). At the mandibular first premolar position, *PAX9* mutation showed a significantly lower percentage of missing teeth (*P* < 0.05). At the maxillary lateral incisor, mandibular lateral incisor, and mandibular central incisor positions, *EDA* mutation showed a significantly higher percentage of missing teeth (*P* < 0.05) (Fig. 2). When PAX9 is mutated the mandibular second and the maxillary first and second molars are frequently absent when EDA is mutated the maxillary lateral and mandibular central and lateral incisors are frequently absent (Fig. 3).

Eleven cases with more than one gene mutation were reported.^19–21^ Some of the subjects presented with a more severe phenotype: two cases with *MSX1* and *PAX9* mutations^19^ (missing teeth number *n* = 15 and 17) compared to family members with a *PAX9* monogenic mutation (missing number *n* = 12), and one case with *WNT10A* and *WNT10B* mutations^20^ (missing number *n* = 22) compared to the heterozygous family members^22^ (average missing teeth number *n* = 13.66).

Ectodermal dysplasia (ED) is the most frequently mentioned syndrome associated with oligodontia. Other mentioned syndromes were Naxos disease (OMIM#601214), Wolf-Hirschhorn syndrome (OMIM#194190), Witkop syndrome (OMIM#189500), and Axenfeld–Rieger syndrome (OMIM # 180500).

## Discussion

The vast majority of tooth agenesis cases in the general population involve only one or two congenitally absent teeth. The absence of one or two permanent teeth is found in 83% of the subjects with dental agenesis.^11^ In a recent study of 1101 individuals, 23 patients showed congenitally missing teeth with a combined total of 34 missing teeth (average = 1.5).^23^ In contrast a systematic review of over a 100 articles identifying the gene/mutation that caused tooth agenesis found the average number of missing teeth per affected individual was over 11, with a range from 1 to 28. Hypodontia (five or less missing teeth) comprised only 11.25% of the total. The rest had oligodontia (at least six missing teeth).^24^ As the average number of missing teeth per patient in genetic studies is far higher than that of the general population (and a mixture of hypodontia and oligodontia cases), genotype-phenotype correlations with respect to an average number of specific teeth or total teeth missing is less likely to be useful for clinicians or geneticists to arrive at a molecular diagnosis in the absence of genetic testing. We, therefore, conducted a study combining new clinical data with a systematic study of oligodontia cases to determine how the frequency of agenesis of specific tooth types varies with respect to the causative gene in oligodontia-only cases.

We observed that the absence of the mand. and max. molars (third molars excluded) is significantly linked to *PAX9* mutation.^25^ PAX9 is a transcription factor belonging to the paired box (PAX) family. A recent study demonstrated that *PAX9*-related oligodontia may be associated with paired domain structural destruction leading to a dominant-negative effect.^26^ Furthermore, a hotspot of *PAX9* mutations is in exon 2, which encodes the paired box DNA binding domain involved in protein–DNA interactions.^19^

The agenesis of anterior teeth is more likely linked to *EDA* mutations.^24,27^ *EDA* encodes ectodysplasin A, a transmembrane protein of the TNF family involved in the EDA/EDAR/NF-κB signaling pathway required for normal embryogenesis, particularly of ectodermal organs including tooth, hair, and skin.^28^ In our review, four cases showed syndromic phenotype including sparse or curly hair, wrinkled skin, or heat intolerance. In this pathway, the EDA receptor (EDAR) binds to its adapter (EDARADD) and forms an EDA-EDAR-EDARADD complex that leads to the downstream activation of NF-κB signaling.^15,29^ Additionally, during tooth development, EDA may regulate the expression of *WNT10A*, *WNT10B*, and *BMP4* via NF-κB.^30^ Interestingly, despite a close relationship between *EDA* and *EDAR*, when these genes are mutated the resulting patterns of oligodontia are distinct.^31^

WNT10A is a member of the Wnt family expressed in the dental epithelium and mesenchyme during odontogenesis. WNT10A binds to the Frizzled transmembrane receptor (G protein-coupled receptor that serves to transduce extracellular signals into cells^32^ and to LRP6 (lipoprotein receptor-related protein 6) co-receptor, leading to the activation of the *Wnt/β-catenin* pathway.^33^ *WNT10A* mutations are predicted to disrupt or reduce *Wnt-mediated* signaling.^34^ resulting in a deficiency of *Wnt/β-catenin* activity and arrest of tooth development.^35^ In contrast, increased expression of *WNT10A* results in upregulation of the β-catenin pathway, which may lead to cancers.^32^

A dosage-dependent pattern has been suggested when it comes to the phenotype caused by *WNT10A* mutations.^36^ Individuals with biallelic *WNT10A* mutations generally present with more severe tooth agenesis when compared to individuals in the same family with defects in a single allele.^37^ Heterozygous *WNT10A* variants resulting in variable penetrance and expressivity has been observed in many studies.^37,38^ These observations are consistent dental phenotypic features observed in the oligodontia subjects of the seven families with *WNT10A* mutations reported here. These features included variations in dental crown and root morphology including microdontia, generalized dental spacing, hypoplastic enamel, conically-shaped teeth, missing disto-palatal cusps on first molars, taurodontic roots, fused roots of maxillary molars, moderate to severe attrition of over-retained primary teeth, and deficient alveolar ridges. The oligodontia phenotype often proved to be a dose effect of combining two defective *WNT10A* alleles from heterozygous parents, sometimes with hypodontia. This was not a strict finding, however. In our seven *WNT10A* families there were six subjects with oligodontia caused by biallelic defects, three caused by single allele defects, and two subjects with biallelic defects that showed a less severe hypodontia phenotype. We searched our WES data paying particular attention to other genes associated with tooth agenesis but could not identify additional sequence variants (genetic modifiers) that correlated with the variations in expressivity.

WNT10B (formerly WNT12) is a protein belonging to the Wnt protein family and encoded by the *WNT10B* gene in humans.^39^ WNT10B is a downstream target of the Eda/Edar/NF-kB signaling pathway during tooth development.^40^ Proper reciprocal interactions between Wnt/β-catenin and Eda/Edar/NF-kB signaling pathways is necessary for tooth development. Therefore, *WNT10B* variant-associated tooth agenesis is hypothesized to be a synergistic result of aberrant Wnt/β-catenin and Eda/Edar/NF-kB signaling pathways.^22^

MSX1 represses transcription of the proximal BMP4 (bone morphogenetic protein 4) promoter. When combined with PAX9, MSX1 acts as a reinforcer of PAX9-induced BMB4 transactivation. BMP4 belongs to the TGF-β superfamily of proteins. It contributes to bone and cartilage development, specifically limb and tooth development and bone fracture repair. Both *MSX1* and *PAX9* are critical to regulate the transition from bud to cap stage of tooth development^41–43^ because these two genes synergistically regulate the expression of BMP4. An in vitro experiment^44^ suggested that the risk for oligodontia may increase when the *PAX9* and *MSX1* gene dosages are reduced. This may explain why cases^19^ with digenic mutations involving *MSX1* and *PAX9* presented with a more severe phenotype. Although Wang et al. suggested that the mechanism of human tooth agenesis caused by *MSX1* mutations may be independent of any synergism with *PAX9*.^45^ Jia, S. et al.^46^ reported a novel mechanism BMP4-MSX1 pathway and OSR2 (protein odd-skipped-related) being implicated in tooth development by antagonistic regulation of secreted molecules involved in the WNT signaling pathway.

KREMEN1 is a high-affinity DKK1 (dickkopf homolog 1) transmembrane receptor that functionally collaborates with DKK1 to block Wnt/β-catenin signaling. Additionally, KREMEN1 is a component of a Wnt/β-catenin membrane signaling complex.^47,48^ In our review, two articles described ten cases with *KREMEN1* mutations resulting in syndromic oligodontia. On average, 9.80 teeth were absent per case. All of the ten cases were missing maxillary lateral incisors, mandibular lateral incisors, and mandibular central incisors.

AXIN2 is a negative feedback regulator of Wnt signaling. AXIN2 plays an important role in the Wnt signaling pathway by regulating β-catenin stability.^49,50^ Lammi et al. demonstrated that AXIN2 regulated tooth formation by showing intensive expression of *Axin2* in the mouse embryos, dental mesenchyme, and enamel knots during odontogenesis.^49^ It has been demonstrated that *AXIN2* mutation carriers show a higher risk of colorectal adenomatous polyposis and/or colorectal cancer.^49^ The phenotype of *AXIN2* oligodontia can be diagnosed noninvasively prior to the detection of colorectal adenomatous polyposis, making it possible to monitor a potential late-onset hereditary cancer risk.^51^

It is important to make clear that the phenotype of the commonly encountered oligodontia causative genes discussed above is tightly linked with the ectoderm-derived organs. There are many less commonly encountered genes linked to oligodontia when mutated also impact basic cellular functions such as transcription or mRNA splicing. Genes such as *BMP4*, *PITX2*, and *NEMO* (*IKBKG*) when mutated have a broad impact on early embryogenesis, therefore, resulting in complex syndromic phenotypes. Because of this review focusing on commonly encountered oligodontia causative genes, it is not possible to review all the causative genes of oligodontia with syndromic presentations. However, the Human Phenotype Ontology, which incorporates both the OMIM and Orphanet curated human disorders is a valuable resource for an in-depth understanding of this complex subject. Furthermore, it is important to keep in mind that literature reports represent a snapshot of cases or conditions in a defined window of time, often the late-onset clinical features and prognosis are less well described.

Oligodontia is a genetic disorder that often presents as an isolated condition but can be part of a syndrome. In our study, ED is the most frequently mentioned syndrome with oligodontia, while *WNT10A* mutations constitute the most frequently determined genetic cause, 53.84%, of the syndromic oligodontia. These findings are consistent with previous reports which established *WNT10A* variants accounting for up to 50% of various ED syndromes with missing teeth.^52^ ED caused by *WNT10A* mutations often include hypohidrotic ectodermal dysplasia (HED), odonto-onycho-dermal dysplasia (OODD) syndrome, and Schopf-Schulz-Passarge syndrome (SSPS). HED is a genetic condition characterized by missing teeth, hypotrichosis, and hypohidrosis. HED is classified into three forms: X-linked hypohidrotic ectodermal dysplasia (XLHED), autosomal recessive hypohidrotic ectodermal dysplasia (ARHED), and autosomal dominant hypohidrotic ectodermal dysplasia (ADHED). OODD is a severe form of autosomal recessive ED manifested by oligodontia, nail dysplasia, keratoderma, hyperhidrosis of the palms and soles, and hyperkeratosis of the skin.^53,54^ SSPS is a rare type of autosomal recessive ED. It is characterized by missing teeth, sparse hair, palmoplantar keratoderma, nail dystrophy, and multiple periocular and eyelid apocrine hidrocystomas.^55^

Apart from missing teeth, there are other features associated with oligodontia: reduced tooth size, altered tooth shape,^56^ taurodontism,^56^ enamel hypoplasia,^57^ ectopic eruption,^58^ over-retained primary teeth,^59^ reduced alveolar development,^60^ tooth surface structure loss,^60^ delayed formations and eruption of permanent teeth,^61^ altered craniofacial morphology,^62^ and increased leeway space.^63^

In our study, the maxillary central incisor was the least affected tooth type. This might be explained by the evolution theory. In 1945 Dalberg used Butler’s Field theory which divided teeth into four morphological fields to explain patterns of tooth agenesis. These fields are molars, premolars, canines, and incisors.^60^ The more medial in each field the tooth was thought to be more genetically stable and less likely to be absent.

## Conclusions

Extensive variations in the patterns of missing teeth were observed among the 393 cases reviewed, highlighting the difficulty and limitation of pathognomonic candidate gene diagnosis for tooth agenesis. In our study, one novel *WNT10A* gene mutation causing oligodontia was identified, expanding the *WNT10A* mutation spectrum, and our analyses identified several new correlations: maxillary and mandibular molar agenesis is most often associated with *PAX9* mutations, maxillary lateral incisor, and mandibular incisors agenesis is most linked to *EDA* mutations, and mandibular first premolar agenesis is least associated with *PAX9* mutations.

Based upon our review, ED is the syndrome most frequently associated with oligodontia. *WNT10A* mutations are the most commonly reported in the genetic etiology for syndromic oligodontia and *PAX9* mutations are the most commonly reported genetic etiology for isolated oligodontia.

Oligodontia is not only a disorder of missing teeth but also a clinical sign of a potentially complex systemic condition. In rare occasions, severe oligodontia can be associated with potentially adverse conditions like hyperplastic polyps with malignant tendencies. Such potential associations make it critical for dentists to understand and become familiar with the correlations between genotype and phenotypes of oligodontia in order to facilitate an accurate diagnosis that may impact the patient’s overall well-being.

## Methods

### Subject recruitment and enrollment

The study protocol and subject consent forms were reviewed and approved by the Ethics Committee at the University of Istanbul and the Institution Review Board at the University of Michigan. Patients with six or more missing teeth (excluding third molars) were recruited. Study explanation, pedigree construction, subject enrollment, clinical examinations, and collection of saliva samples were completed under the proper consenting procedure specified in the study protocols. Available subjects were evaluated clinically and radiographically. The ectodermal organs reviewed include hair (scalp and facial hair), nail (smooth or dysplastic), and skin (facial and hand skin dryness). We asked subjects to describe their toenails, signs of dry skin (redness, roughness, and size of the dry patch if present), excessive sweating or limited sweating, and dryness of eyes. Family members unavailable for evaluation received phone interviews.

### Whole-exome sequencing and bioinformatics analysis

Genomic DNA was isolated from non-stimulated saliva samples following the manufacturer’s protocol (Norgen Biotek Corp., Thorold, ON, Canada). Samples from the parents and proband of each family were selected for whole-exome sequencing (WES), and DNA samples from all other family members were used for segregation analyses. Trio DNA samples following the initial quality control were submitted to Johns Hopkins Center for Inherited Disease Research (CIDR, Baltimore, MD) for WES. GATK’s reference confidence model workflow was used to perform joint sample genotyping and all variants were annotated using VarSeq (Golden Helix, Bozeman, MT). Following the comparisons between the affected and unaffected individuals, a list of prioritized variants was then subjected to segregation analysis.

### Segregation analyses using Sanger sequencing

The prioritized sequence variations and their segregation with the oligodontia phenotype within each family was confirmed by Sanger sequencing. PCR primers were designed to bracket the candidate variant and reactions were conducted following established protocols.^56^ Chromatograms of all participating subjects from the ten study families are reviewed and complied as in Fig. S11.

### Phenotypic analyses

The number and type of missing, present, and extracted teeth was determined by reviewing available dental records including clinical photos and radiographic images. Two independent clinical specialists (a pediatric dentist and an orthodontist), appraised all available records for each individual recruited in the study and determined which teeth were present, absent, and/or extracted. When the assessments were not in agreement, the two specialists reviewed the images together to reach a consensus.

### Sources of data and search strategy

Following standard literature review procedures, a search of the OMIM database and recent publications was conducted on July 3, 2021 to determine genes associated with oligodontia. The genes identified from this search included: *MSX1, EDA, PAX9, WNT10B, WNT10A, LRP6, EDARADD, EDAR, POLR3A, AXIN2, SMOC2, LTBP3, DSP, SATB2, BCOR, ATP6V1B2, SLC25A24, CDH1, KCNJ2, HUWE1, PPP1R15B, PIK3C2A, PDE3A, UBR1, IKBKB, EVC2, KIAA1279, SRD5A3, DVL1, MESD*, *TSPEAR*, *COL1A1*, *CREB3L1*, and *KREMEN1*. We then conducted a search on July 3, 2021 of PubMed and Web of Science for articles published in English between January 1, 1992, and June 30, 2021, using the above-mentioned “gene name” and “oligodontia” as key words. Based upon the review of study key words, title, and abstract, relevant studies were selected for a comprehensive systematic review. A list of 96 articles reviewed in this study is presented at the end of the supplementary document. Additionally, a brief summary of selected findings from those 96 articles is compiled and presented in the supplementary Table S1. Five genes with the largest number of oligodontia cases reported in the literature were reviewed in-depth.

### Eligibility criteria

Articles were included in the review if they met the following criteria: written in English-language, the subjects were humans, included a genetic mutation from one of the above-mentioned candidate genes, included the patients’ dental radiographs allowing confirmation of oligodontia and assessment of dental phenotype, the number of missing teeth equaled to or greater than six (excluding third molars), but not edentulous. Cases with more than one genetic mutation were excluded. A flow diagram of the search strategy is presented in Fig. S1.

### Data extraction and synthesis

Specific data including genetic mutation, phenotypic diagnosis, mode of inheritance, case demographics, and non-dental phenotype were compiled from all cases that met the above-described criteria. Missing teeth locations were assessed by the same specialists mentioned previously using panoramic radiographs. Cases were grouped for qualitative/descriptive analysis according to their causative genes. The mean number of missing permanent teeth for each causative gene group and the frequency of tooth absence at each tooth position were calculated. Each causative gene group with more than one reported case was included for such analyses. Mean number and missing teeth percentage were calculated using Microsoft Excel (Version 16.0.5110.100; 2016 Microsoft Corporation). Statistical analysis was performed using the *χ*2 test (IBM SPSS Statistics 26) and Prism 8 (GraphPad Prism 8.1.2, Macintosh Version, GraphPad Software, San Diego, CA, USA). The value of *p* < 0.05 was considered statistically significant.

## Supplementary information

Oligodontia Supplemental Data Revised
