# Correction: Analyses of oligodontia phenotypes and genetic etiologies

**DOI:** 10.1038/s41368-021-00141-5

**Published:** 2021-11-12

**Authors:** Mengqi Zhou, Hong Zhang, Heather Camhi, Figen Seymen, Mine Koruyucu, Yelda Kasimoglu, Jung-Wook Kim, Hera Kim-Berman, Ninna M. R. Yuson, Paul J. Benke, Yiqun Wu, Feng Wang, Yaqin Zhu, James P. Simmer, Jan C-C. Hu

**Affiliations:** 1 Dental Research Laboratory, University of Michigan School of Dentistry, Ann Arbor, MI USA; 2 Department of Second Dental Center, Shanghai Ninth People’s Hospital, Shanghai Jiao Tong University School of Medicine; College of Stomatology, Shanghai Jiao Tong University; National Center for Stomatology; National Clinical Research Center for Oral Diseases; Shanghai Key Laboratory of Stomatology, Shanghai, China; 3 Department of General Dentistry, Shanghai Ninth People’s Hospital, Shanghai Jiao Tong University School of Medicine; College of Stomatology, Shanghai Jiao Tong University; National Center for Stomatology; National Clinical Research Center for Oral Diseases; Shanghai Key Laboratory of Stomatology, Shanghai, China; 4 Orthodontic and Pediatric Dentistry, University of Michigan School of Dentistry, 1011N. University Ave, Ann Arbor, MI USA; 5 Mott Children’s Health Center 806 Tuuri Place, Flint, MI USA; 6 Department of Pedodontics, Faculty of Dentistry, Istanbul University, Istanbul, Turkey; 7 Department of Molecular Genetics & Dental Research Institute School of Dentistry, Seoul National University, Seoul, Korea; 8 Department of Pediatric Dentistry & Dental Research Institute School of Dentistry, Seoul National University, Seoul, Korea; 9 Department of Paediatric Dentistry, Women’s and Children’s Hospital, North Adelaide, SA Australia; 10 Department of Medical Genetics, Joe DiMaggio Children’s Hospital, Hollywood, FL USA; 11 Department of Oral Implantology, Shanghai Ninth People’s Hospital, Shanghai Jiao Tong University School of Medicine; College of Stomatology, Shanghai Jiao Tong University; National Center for Stomatology; National Clinical Research Center for Oral Diseases; Shanghai Key Laboratory of Stomatology, Shanghai, China

**Keywords:** Dental diseases, Disease genetics

Correction to: *International Journal of Oral Science* 10.1038/s41368-021-00135-3, published online 30 September 2021

Following publication of this article,^1^ it is noticed Table 2 in this article needs revision.

**Table 2:**
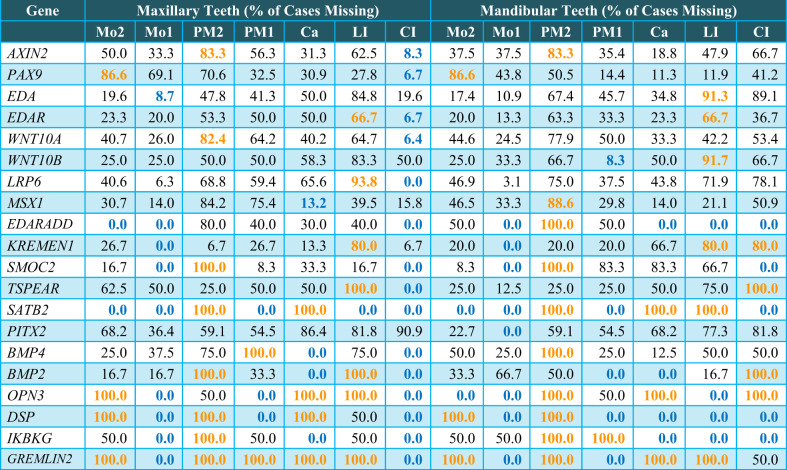
Frequencies of tooth absence (in percentage) among the study cases affected by *PAX9*, *MSX1*, *WNT10A*, *WNT10B*, *AXIN2*, *EDA*, *EDAR*, *EDARADD*, *LRP6*, *KREMEN1*, *SMOC2*, and *PITX2* gene mutations

Please note that the same type of tooth absent in the right and left arches were pooled together for the calculation. Numbers in gold color represent the highest frequency and numbers in blue color represent the lowest frequency of tooth type absence in each causative gene *Ca* canine; *CI* central incisor; *LI* lateral incisor; *Mo* molar; *PM* premolar.

The correct Table 2 is provided in this Correction.

The original article has been updated.
