# Supplementary material for: Analyses of oligodontia phenotypes and genetic etiologies
Source: Int J Oral Sci. 2021 Sep 30;13:32. doi: 10.1038/s41368-021-00135-3 (PMC8484616; doi:10.1038/s41368-021-00135-3)
Supplement: Supplementary file 1 — Oligodontia Supplemental Data Revised [file 41368_2021_135_MOESM1_ESM.pdf]

Analyses of Oligodontia Phenotypes and Genetic Etiologies

Mengqi Zhou[1,2,3], Hong Zhang[1], Heather Camhi[4,5], Figen Seymen[6], Mine Koruyucu[6], Yelda Kasimoglu[6], Jung-Wook Kim[7,8], Hera Kim-Berman[4], Ninna M. R. Yuson[9], Paul J. Benke[10], Yi-Qun Wu[2], Feng Wang[11], Ya-Qin Zhu[3], James P. Simmer[1] and Jan C-C. Hu[1].

1. Dental Research lab, University of Michigan School of Dentistry, 1210 Eisenhower Place, Ann Arbor, Michigan, USA.
2. Department of Second Dental Center, Shanghai Ninth People's Hospital, Shanghai Jiao Tong University School of Medicine; College of Stomatology, Shanghai Jiao Tong University; National Center for Stomatology; National Clinical Research Center for Oral Diseases; Shanghai Key Laboratory of Stomatology. No.639 Zhizaoju Road, Huangpu District, Shanghai, China
3. Department of General Dentistry, Shanghai Ninth People's Hospital, Shanghai Jiao Tong University School of Medicine; College of Stomatology, Shanghai Jiao Tong University; National Center for Stomatology; National Clinical Research Center for Oral Diseases; Shanghai Key Laboratory of Stomatology. No.639 Zhizaoju Road, Huangpu District, Shanghai, China
4. Orthodontic and Pediatric Dentistry, University of Michigan School of Dentistry, 1011 N. University Ave. Ann Arbor, Michigan, USA.
5. Mott Children's Health Center 806 Tuuri Place, Flint, Michigan, USA.
6. Department of Pedodontics, Faculty of Dentistry, Istanbul University, Istanbul, Turkey.
7. Department of Molecular Genetics & Dental Research Institute School of Dentistry, Seoul National University, Seoul, Korea.
8. Department of Pediatric Dentistry & Dental Research Institute School of Dentistry, Seoul National University, Seoul, Korea.
9. Department of Paediatric Dentistry, Women's and Children's Hospital, 72 King William Rd, North Adelaide, SA 5006, Australia.
10. Department of Medical Genetics, Joe DiMaggio Children's Hospital, 1150 N 35th Avenue, Suite 490. Hollywood, FL 33021, USA
11. Department of Oral Implantology, Shanghai Ninth People's Hospital, Shanghai Jiao Tong University School of Medicine; College of Stomatology, Shanghai Jiao Tong University; National Center for Stomatology; National Clinical Research Center for Oral Diseases; Shanghai Key Laboratory of Stomatology

Contents:

Figure S1. Flow chart of the article selection and review process.

Figure S2. Oligodontia Family 2 from Turkey with Compound Heterozygous *WNT10A* 3 defects c.433G>A, p.Val145Met and c.682T>A, p.(Phe228Ile).

Figure S3. Oligodontia Family 3 from Turkey with Homozygous *WNT10A* defect: c.433G>A, p.(Val145Met).

Figure S4. Oligodontia Family 4 with Compound Heterozygous *WNT10A* Defects c.318C>G, p.(Asn106Lys) and c.682T>A, p.(Phe228Ile).

Figure S5. Oligodontia Family 5 with the Heterozygous *WNT10A* defect: c.682T>A, p.(Phe228Ile).

Figure S6. Oligodontia Family 6 with the Heterozygous *WNT10A* defect: c.321C>A, p.(Cys107*).

Figure S7. Oligodontia Family 7 with the Homozygous *WNT10A* defect: c.682T>A, p.(Phe228Ile).

Figure S8. Oligodontia Family 8 with the Heterozygous *EDAR* defect: c.581C>T, p.(Thr194Ile).

Figure S9. Oligodontia Family 9 Heterozygous with the *LRP6* defect: c.1003C>T, p.(Arg335*).

Figure S10. Oligodontia Family 10 Heterozygous with the *LRP6* defect: c.2247G>T, p.(Cys916Phe).

Figure S11. Chromatograms of all participating subjects from 10 study families.

Table S1. List of articles reviewed in this study with brief summaries of their specific findings.

List of Articles Analyzed in this Study.

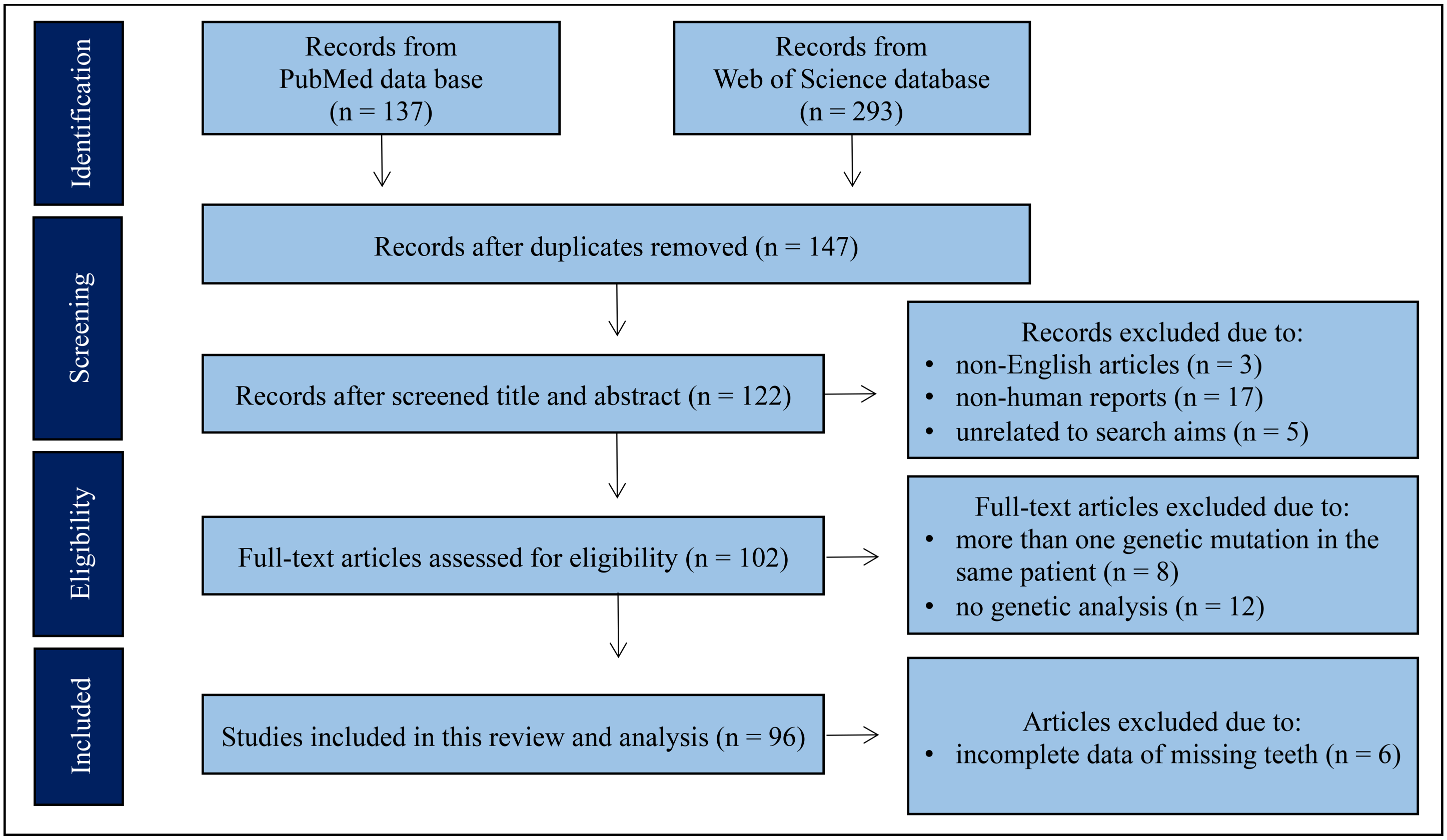

Figure S1. Flow chart of the article selection and review process.

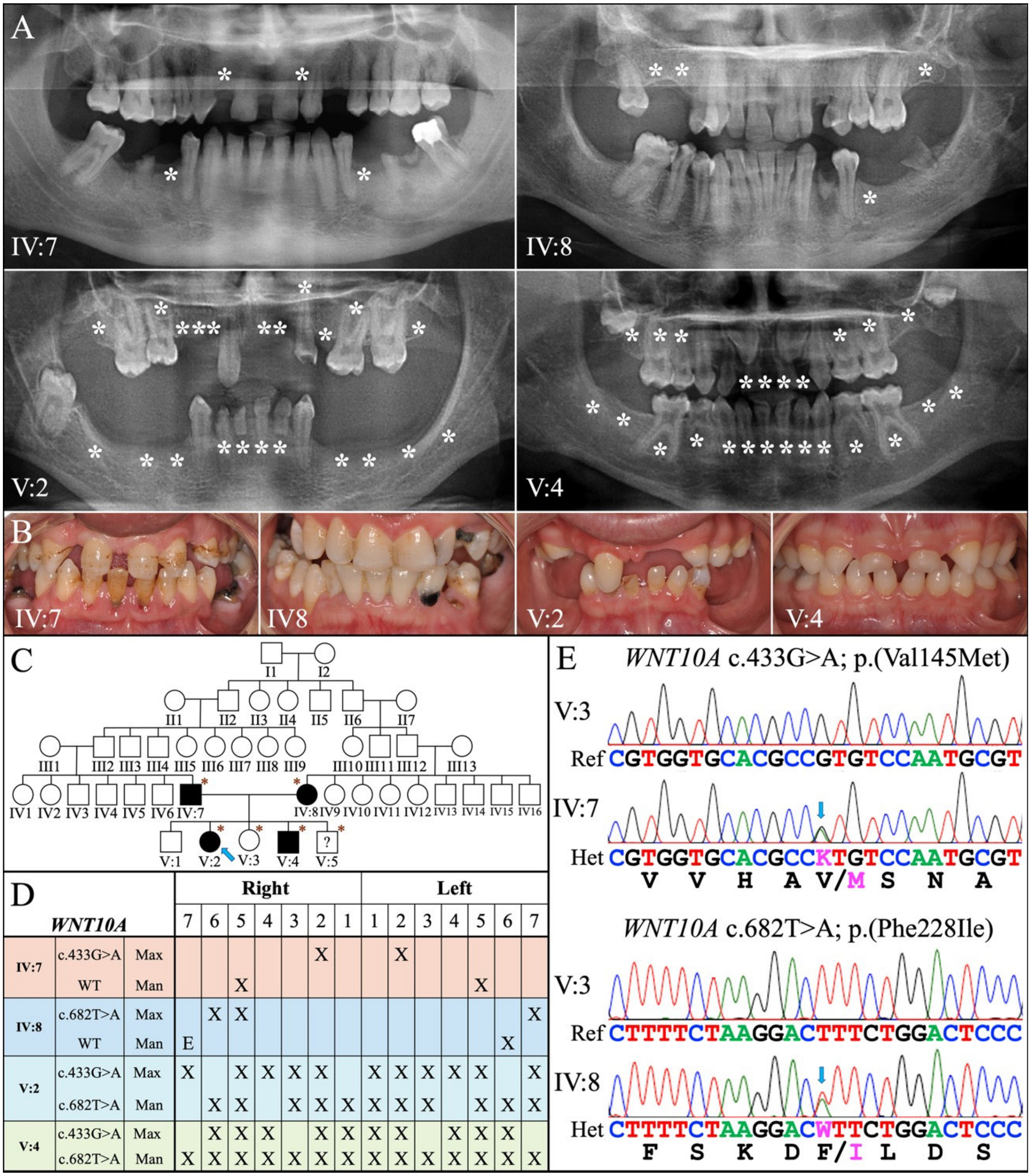

D	*WNT10A*		Right 7	Right 6	Right 5	Right 4	Right 3	Right 2	Right 1	Left 1	Left 2	Left 3	Left 4	Left 5	Left 6	Left 7
IV:7	c.433G>A	Max						X			X					
	WT	Man			X									X		
IV:8	c.682T>A	Max		X	X											X
	WT	Man	E												X	
V:2	c.433G>A	Max	X		X	X	X	X		X	X	X	X	X		X
	c.682T>A	Man		X	X		X	X	X	X	X	X		X	X	X
V:4	c.433G>A	Max		X	X	X		X	X	X	X		X	X	X	
	c.682T>A	Man	X	X	X	X	X	X	X	X	X	X	X	X	X	X

Figure S2. Oligodontia Family 2 from Turkey with Compound Heterozygous *WNT10A* defects c.433G>A, p.(Val145Met) and c.682T>A, p.(Phe228Ile). ***A:*** Panoramic radiographs with sites of tooth agenesis marked by stars. When the radiographs were taken, subjects IV:7 and IV:8 were adults and subjects were V:2 (16y) and V:4 (8y). ***B:*** Oral photographs of subjects. ***C:*** Pedigree of Family 2. Asterisks mark the 6 subjects who participated in the study. Their genotypes are IV:7 +/-, IV:8 +/-, V:2 -/-, V:3 +/+, V:4 -/-, and V:5 +/-. Affection statuses of non-participants areunknown. ***D:*** Summary chart of missing teeth (X, agenesis; E, extracted). The heterozygous parents (IV:7 and IV:8) were both missing 4 teeth. Offspring with biallelic *WNT10A* defects (V:2 and V:4) exhibited agenesis of 22 and 24 teeth, respectively and were included in the oligodontia analyses. Subject V:5 (3y) was heterozygous for the p.(Val145Met) variation only and was too young to determine affection status. Subjects V:2 and V:4 were included in the oligodontia analysis. ***E:*** *WNT10A* chromatograms showing that the father (IV:7) and mother (IV:8) were heterozygous (Het) for the *WNT10A* variations c.433G>A, p.(Val145Met) and c.682T>A, p.(Phe228Ile), respectively. Unaffected subject V:3 was homozygous for the *WNT10A* reference sequence (Ref). K = A or G; W = A or T. Sequences altered by mutation are in magenta. The NCBI reference sequence designations for these variants are NG_012179.1: g.14508G>A; NG_012179.1(WNT10A_v001): c.433G>A, p.(Val145Met) and NG_012179.1: g.14757T>A; NG_012179.1(WNT10A_v001): c.682T>A, p.(Phe228Ile).

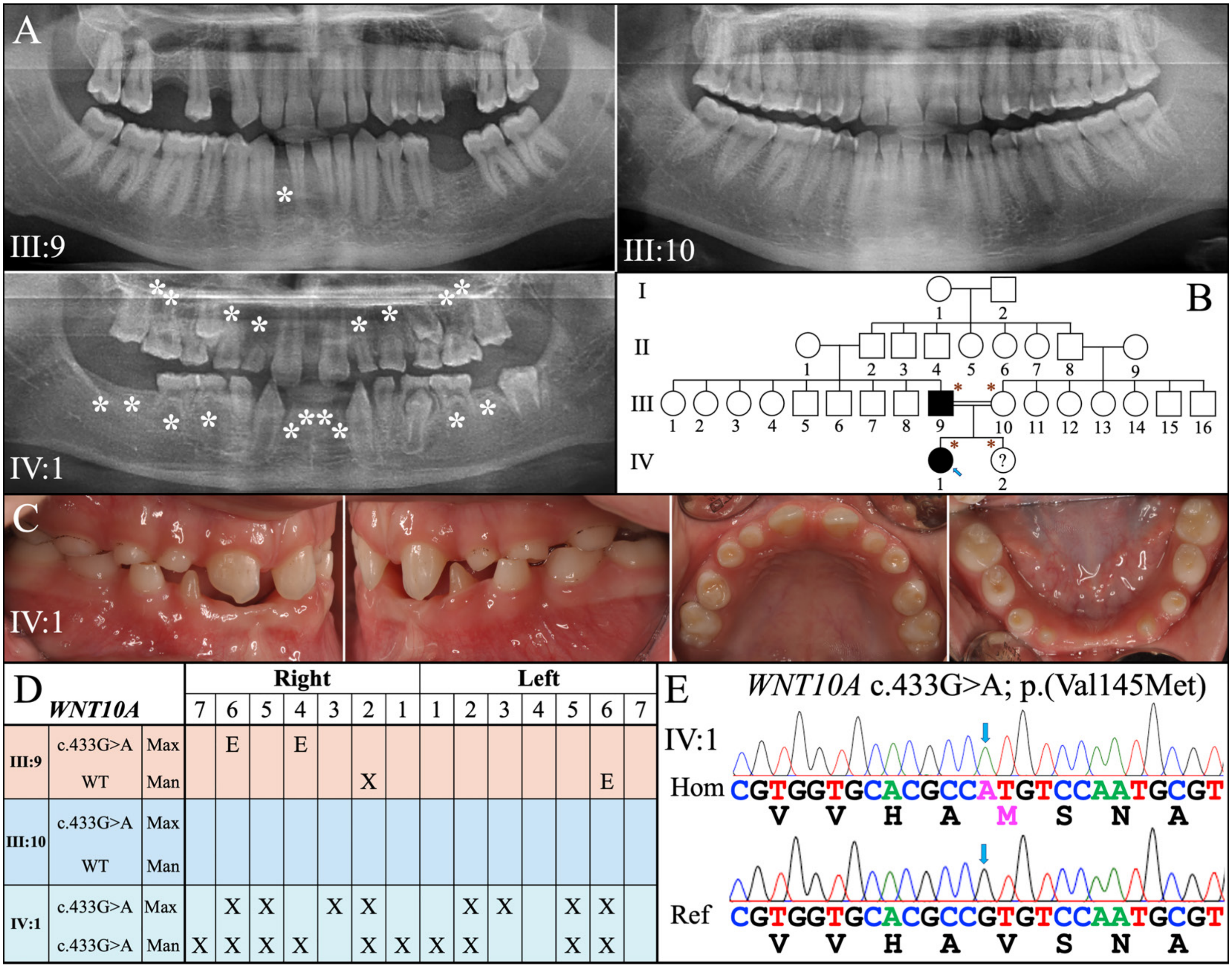

WNT10A			Right 7	Right 6	Right 5	Right 4	Right 3	Right 2	Right 1	Left 1	Left 2	Left 3	Left 4	Left 5	Left 6	Left 7
III:9	c.433G>A	Max		E		E										
	WT	Man						X							E	
III:10	c.433G>A	Max														
	WT	Man														
IV:1	c.433G>A	Max		X	X		X	X			X	X		X	X	
	c.433G>A	Man	X	X	X	X		X	X	X	X			X	X	

Figure S3. Oligodontia Family 3 from Turkey with Homozygous *WNT10A* defect: c.433G>A, p.(Val145Met). ***A:*** Panoramic radiographs with sites of tooth agenesis marked by stars. When the radiographs were taken, the parents (III:9 and III:10) were adults and the offspring (IV:1 and IV:2) were ages 10 and 4, respectively. ***B:*** Pedigree of Family 3. Asterisks mark the 4 subjects who participated in the study. Their genotypes are III:9 +/-, III:10 +/-, IV:1 -/-, and IV:2 +/-. Affection statuses of non-participants are unknown. ***C:*** Oral photographs of proband (IV:1) showing mis-shapened teeth and attrition of retained primary teeth. ***D:*** Summary chart of missing teeth (X, agenesis; E, extracted). The proband exhibited agenesis of 18 permanent teeth and was included in the oligodontia data analysis. Subject IV:2 was heterozygous for the *WNT10A* defect (not shown) and too young to determine affection status. ***E:*** The *WNT10A* chromatogram shows that the proband (IV:I) was homozygous for the *WNT10A* defect. The NCBI reference sequence designation for this *WNT10A* variant is NG_012179.1: g.14508G>A; NG_012179.1 (WNT10A_v001): c.433G>A, p.(Val145Met).

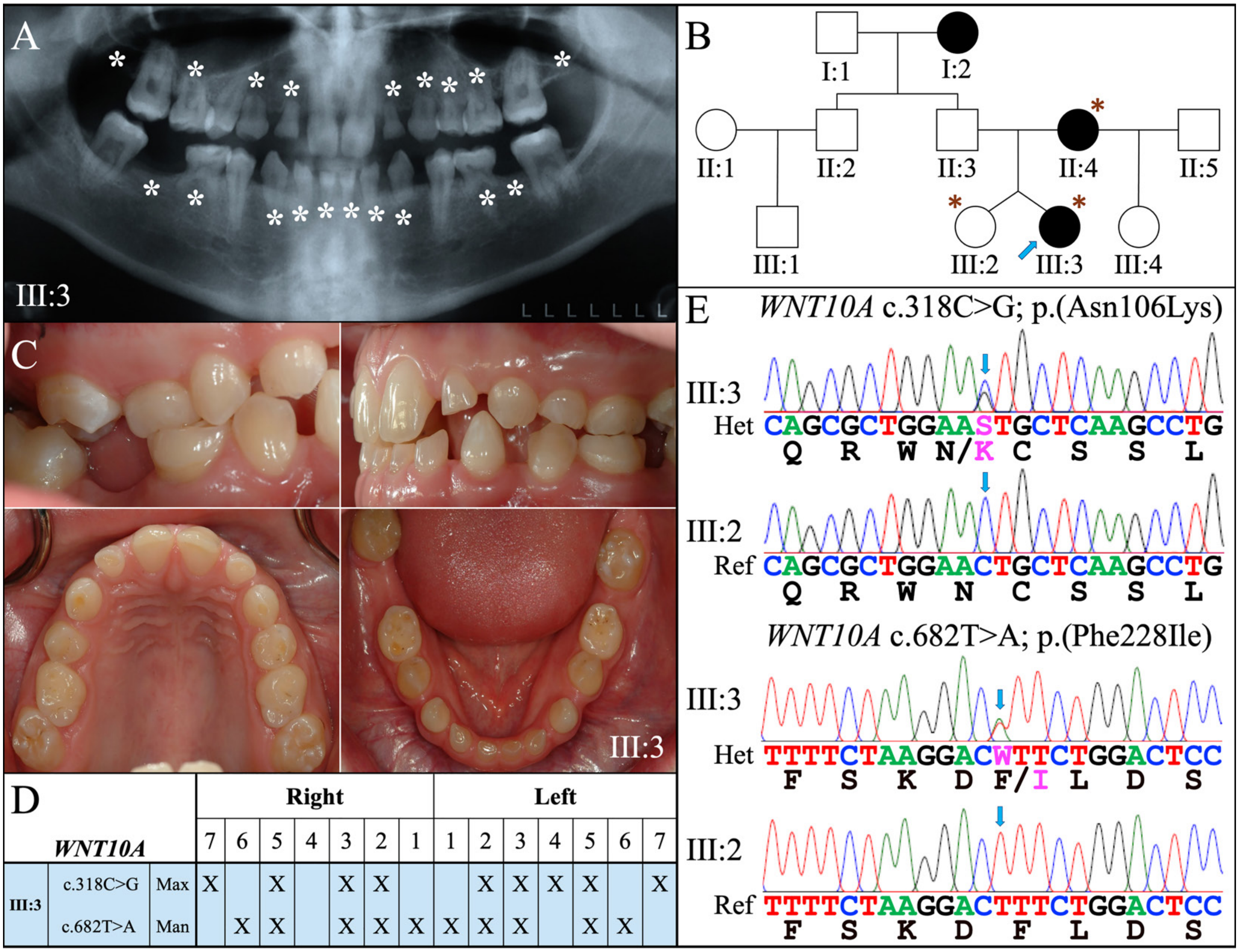

Figure S4. Oligodontia Family 4 with Compound Heterozygous *WNT10A* Defects c.318C>G, p.(Asn106Lys) and c.682T>A, p.(Phe228Ile). ***A:*** Panoramic radiograph of the proband (III:3) with sites of tooth agenesis marked by stars. No radiographs were available for the affected mother (II:4) who reported to have two maxillary lateral incisors absent. ***B:*** Pedigree of Family 4. Asterisks mark subjects recruited in this study. Their genotypes are II:4 +/-, III:2 +/+, and III:3 -/-. ***C:*** Oral photographs of the proband (III:3) who lacked ***D:*** Summary chart showing the sites of tooth agenesis (X). The proband (III:3) exhibited tooth agenesis of 18 permanent teeth and was included in the data analysis. ***E:*** The *WNT10A* chromatograms show that the proband (III:3) was a compound heterozygote for the *WNT10A* c.318C>G, p.(Asn106Lys) and c.682T>A, p.(Phe22Ile) defects, whereas her fraternal twin sister shows the reference sequence at both variant sites. Sequences altered by mutation are in magenta. S = G or C; W = A or T. The NCBI reference sequence designations for these *WNT10A* variants are NG_012179.1: g.6833C>G; NG_012179.1 (WNT10A_v001): c.318C>G, p.(Asn106Lys) and NG_012179.1: g.14757T>A; NG_012179.1(WNT10A_v001): c.682T>A, p.(Phe228Ile).

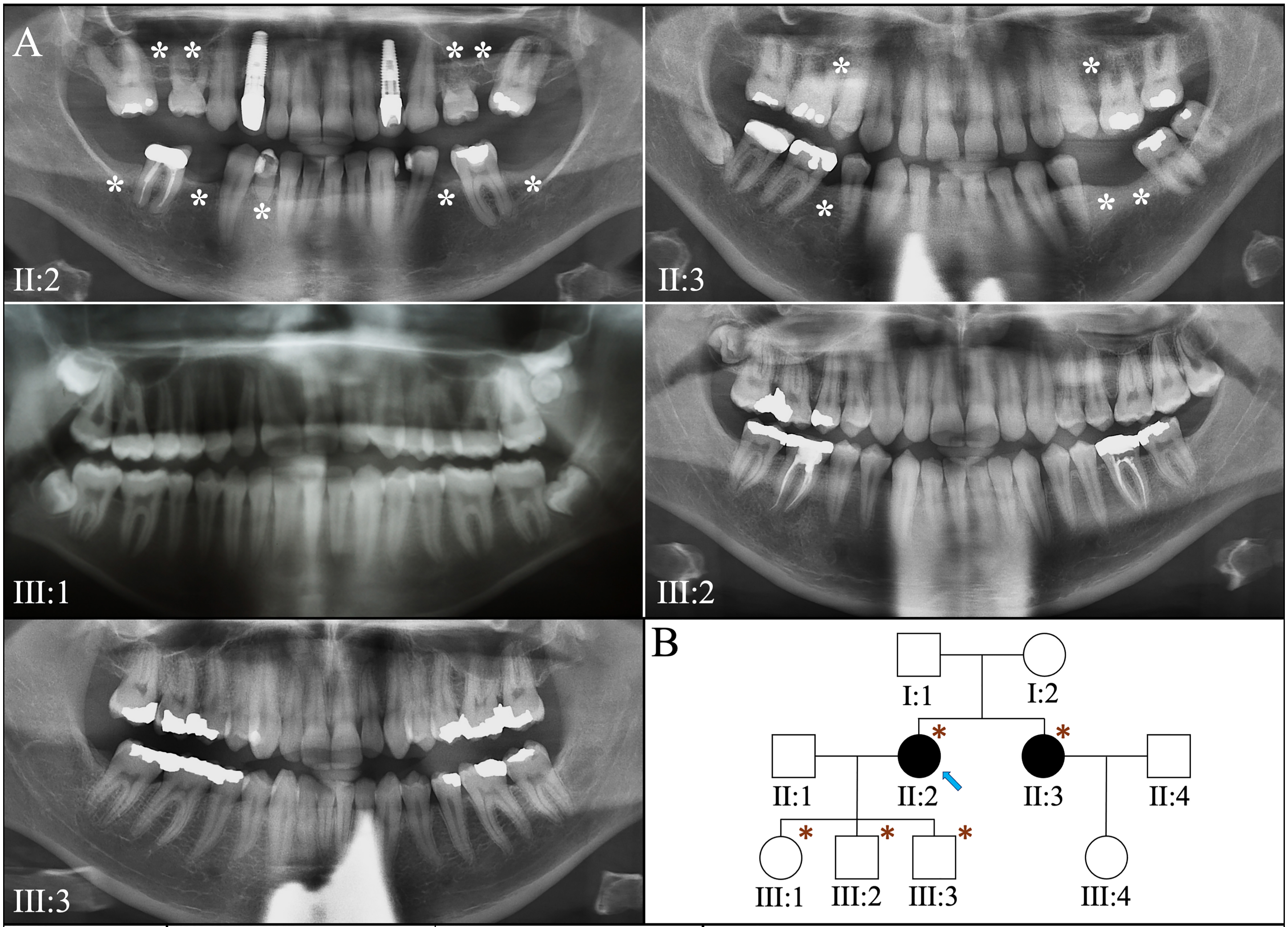

C

	WNT10A		Right 7	Right 6	Right 5	Right 4	Right 3	Right 2	Right 1	Left 1	Left 2	Left 3	Left 4	Left 5	Left 6	Left 7
**II:2**	c.682T>A	Max		X	X		E					E		X	X	
	WT	Man	X		X		X							X		X
**II:3**	c.682T>A	Max			X									X		
	WT	Man			X									X	X	
**III:1**	WT	Max														
	WT	Man														
**III:2**	c.682T>A	Max														
	WT	Man														
**III:3**	c.682T>A	Max														
	WT	Man														

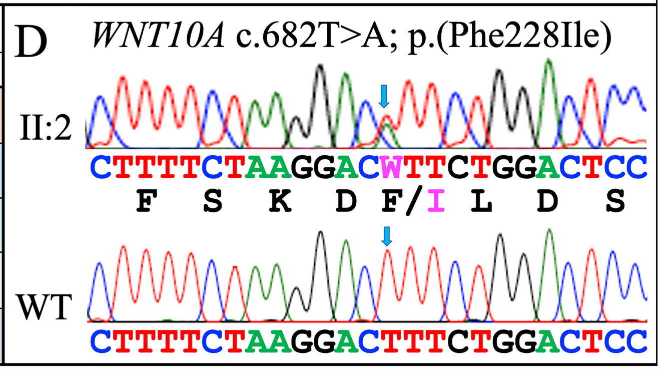

Figure S5. Oligodontia Family 5 with the Heterozygous *WNT10A* defect: c.682T>A, p.(Phe228Ile). ***A:*** Panoramic radiographs of Family 5 with sites of tooth agenesis marked by stars. ***B:*** Pedigree of Family 5. Asterisks mark the 5 subjects recruited in this study. Their genotypes are II:2 +/-, II:3 +/-, III:1 +/+, III:2 +/-, and III:3 +/-. ***C:*** Summary chart of missing teeth (X, agenesis;E, extracted). The proband (II:2) exhibited agenesis of 9 permanent teeth and was included in the oligodontia data analysis. ***D:*** The *WNT10A* chromatogram shows the proband (II:2) was heterozygous for the c.682T>A, p.(Phe228Ile) defect, as were subjects II:3 and III:3 (not shown). Sequences altered by mutation are in magenta. W = A or T. The NCBI reference sequence designations for this *WNT10A* variant are NG_012179.1: g.14757T>A; NG_012179.1(WNT10A_v001): c.682T>A, p.(Phe228Ile).

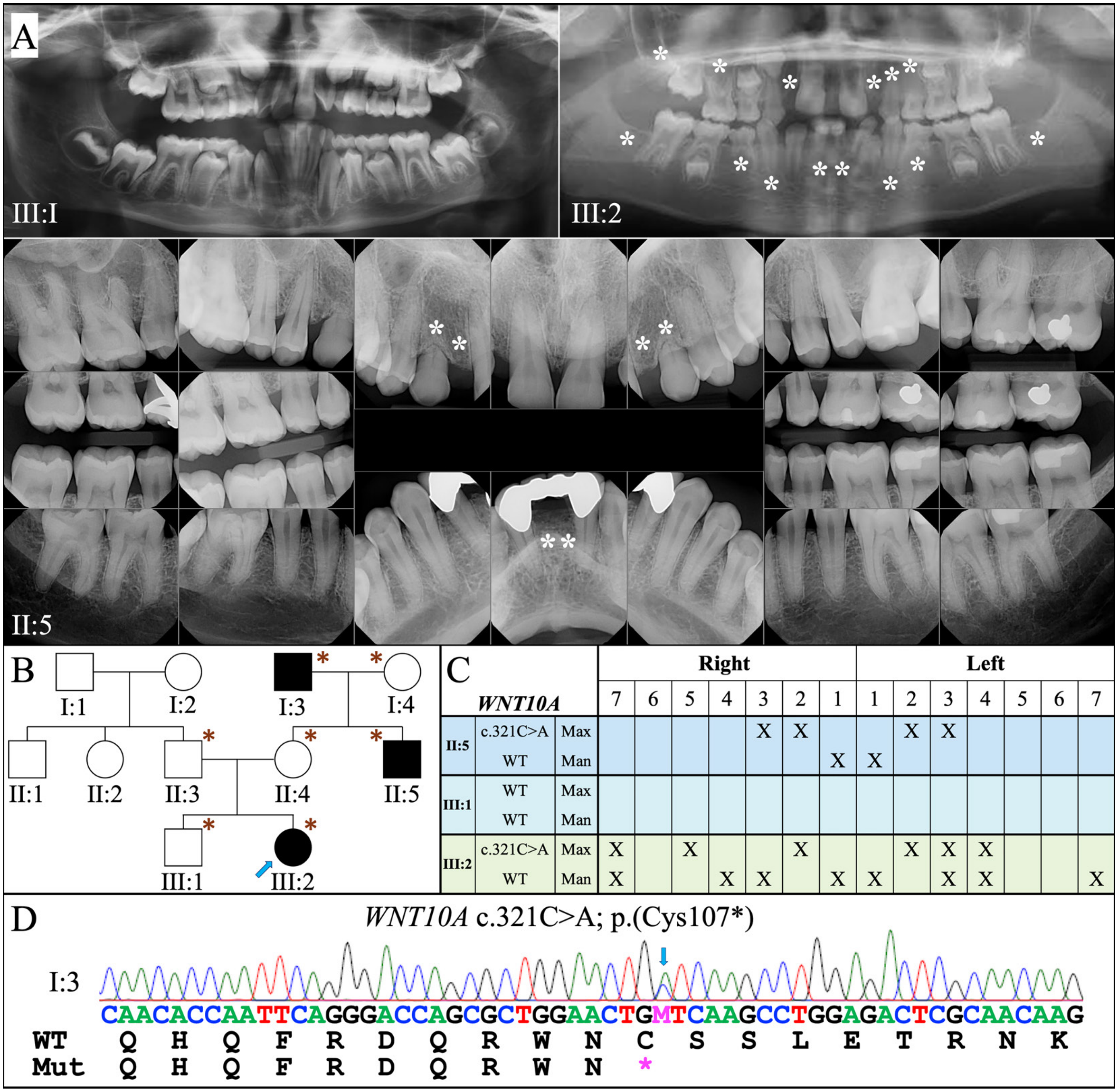

Figure S6. Oligodontia Family 6 with the Heterozygous *WNT10A* defect: c.321C>A, p.(Cys107*). ***A:*** Panoramic radiographs of Family 6 (III:1, 10 years old; and III:2, 7 years old) and anterior periapical radiographs of subject II:5 (adult) with sites of tooth agenesis marked by stars. ***B:*** Pedigree of Family 6. Asterisks mark the 7 subjects recruited in this study. Their genotypes are I:3 +/-, I:4 +/+, II:3 +/+, II:4 +/-, II:5 +/-, III:1 +/+ and III:2 +/- ***C:*** Summary chart showing the sites of tooth agenesis (X). The proband (III:2) lacked 14, and subject II:5 lacked 6 permanent teeth and both were included in the data analysis. ***D:*** Chromatogram from subject I:3 showing the heterozygous *WNT10A* c.321C>A, p.(Cys107*) defect that was also identified in subjects II:4, II:5, and the proband (III:2), although the number of missing teeth on subjects I:3 and II:4 could not be ascertained . Subjects I:4 and II:3 were wild-type for this *WNT10A* defect (not shown). Sequences altered by mutation are in magenta.M = A or C. The NCBI reference sequence designations for this *WNT10A* variant is NG_012179.1: g.6836C>A; NG_012179.1(WNT10A_v001): c.321C>A; NG_012179.1(WNT10A_i001): p.(Cys107*).

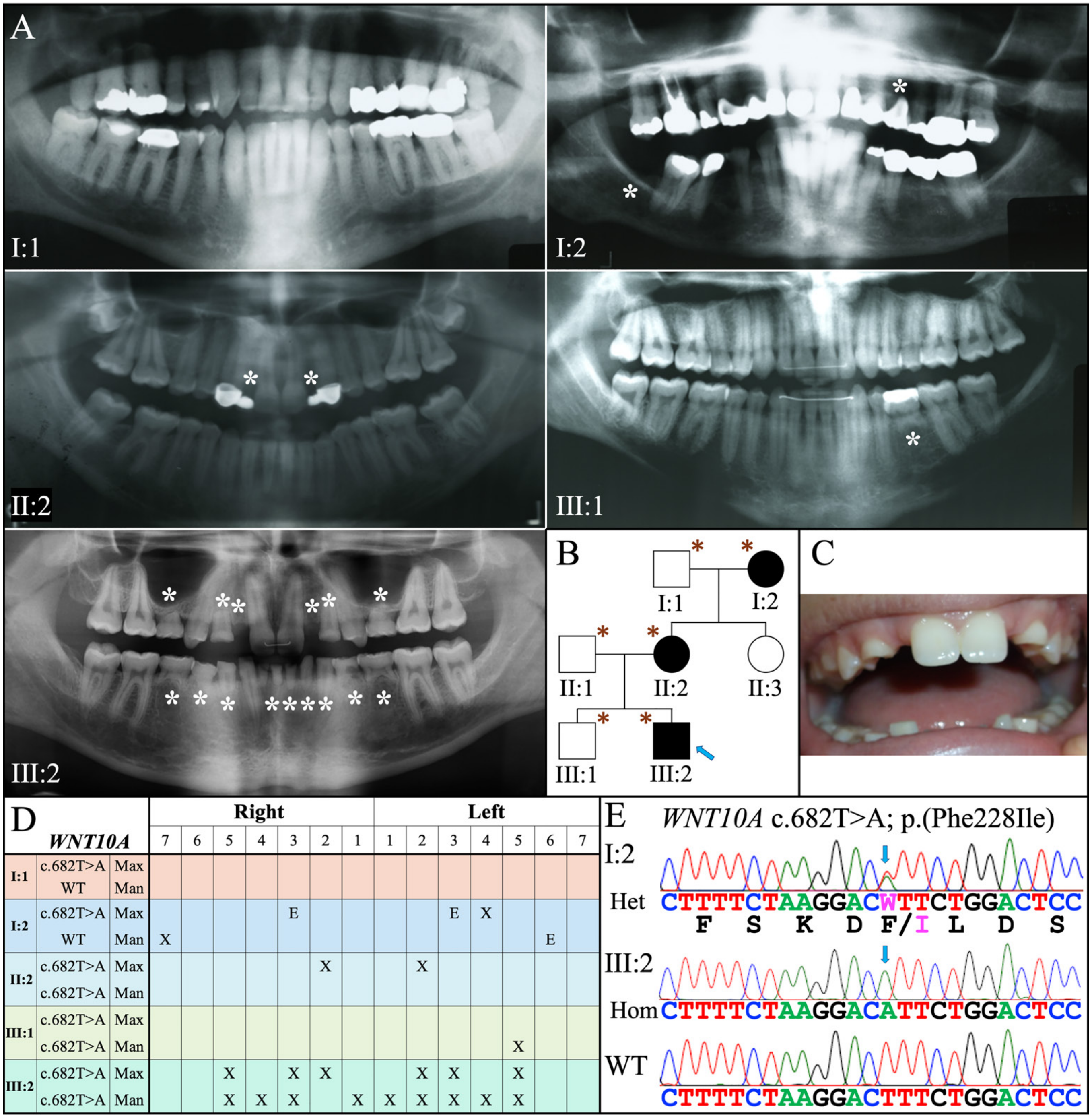

D	*WNT10A*		Right 7	Right 6	Right 5	Right 4	Right 3	Right 2	Right 1	Left 1	Left 2	Left 3	Left 4	Left 5	Left 6	Left 7
I:1	c.682T>A	Max														
	WT	Man														
I:2	c.682T>A	Max					E					E	X			
	WT	Man	X												E	
II:2	c.682T>A	Max						X			X					
	c.682T>A	Man														
III:1	c.682T>A	Max														
	c.682T>A	Man												X		
III:2	c.682T>A	Max			X		X	X			X	X		X		
	c.682T>A	Man			X	X	X		X	X	X	X	X	X		

Figure S7. Oligodontia Family 7 with the Homozygous *WNT10A* defect: c.682T>A, p.(Phe228Ile). ***A:*** Panoramic radiographs of Family 7. A star marks the location of each absent (undeveloped) permanent tooth. ***B:*** Pedigree of Family 7. Asterisks mark subjects recruited in this study. Their genotypes are I:1 +/-, I:2 +/-, II:1 +/-, II:2 -/-, III:1 -/-, and III:2 -/-. ***C:*** Oral photo of proband (III:2) showing a lack of contour on the central incisors and attrition of the retained primary teeth. ***D:*** Summary chart of missing teeth (X, agenesis; E, extracted). Despite the fact that subjects I:1 and II:1 were heterozygous, and subjects I:2, III:1, and III:2 were homozygous for the c.682T>A, p.(Phe228Ile) defect, only the proband (III:2) showed oligodontia agenesis of 15 permanent teeth) and was included in the data analysis. ***E:*** Chromatograms showing the heterozygous (I:2), homozygous (III:2), and wild-type (II:1) sequences for the c.682T>A, p.(Phe228Ile) variation. Subjects II:1 and III:2 also carried a heterozygous *EDARADD* variation NM_145861.4: c.308C>T; p.(Ser103Phe) (rs114632254), which was previously described as a functional variant. [Salvi, A. et al.Mutation analysis by direct and whole exome sequencing in familial and sporadic tooth agenesis. *Int J Mol Med* **38**, 1338-1348, (2016)]. The NCBI reference sequence designations for this *WNT10A* variant are NG_012179.1: g.14757T>A; NG_012179.1(WNT10A_v001): c.682T>A, p.(Phe228Ile).

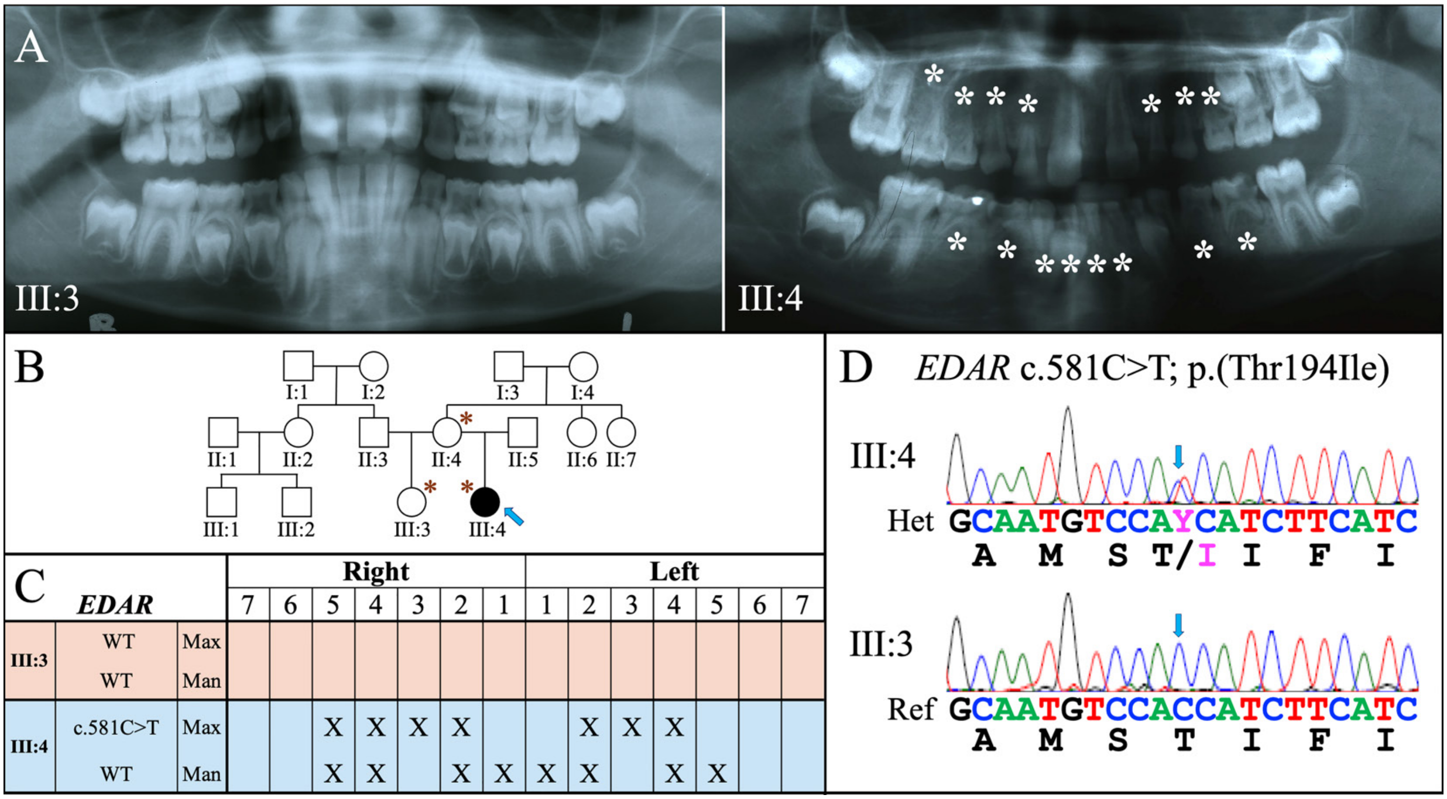

C	*EDAR*		Right 7	Right 6	Right 5	Right 4	Right 3	Right 2	Right 1	Left 1	Left 2	Left 3	Left 4	Left 5	Left 6	Left 7
**III:3**	WT	Max														
	WT	Man														
**III:4**	c.581C>T	Max			X	X	X	X			X	X	X			
	WT	Man			X	X		X	X	X	X		X	X		

Figure S8. Oligodontia Family 8 with the Heterozygous *EDAR* defect: c.581C>T, p.(Thr194Ile). ***A:*** Radiographs of III:3 and III:4 in Family 8. At the time when the radiographs were taken, subjects III:3and III:4 were age 9 years 7 months and 8 years 2 months, respectively. ***B:*** Pedigree of Family 8. Asterisks mark the 3 subjects recruited in this study. Their genotypes are II:4 +/+, III:3 +/+, and III:4 +/-. ***C:*** Summary chart showing the sites of tooth agenesis (X) for subjects III:3 and III:4. The proband (III:4) lacked 15 permanent teeth and was included in the data analysis. ***D:*** Chromatograms of subjects III:4 and III:3. Sequences altered by mutation are in magenta. Y = T or C. The *EDAR* chromatograms showed that subject III:4 was heterozygous for the *EDAR* sequence variation and subject III:3 was wild-type (*EDAR* sequence was identical to the reference). The mother (II:4) was also wild-type (not shown). No other *EDAR* sequencevariations were observed. The NCBI reference sequence designations for this *EDAR* variant are NG_008257.1: g.83352C>T; NG_008257.1(EDAR_v001): c.581C>T, p.(Thr194Ile).

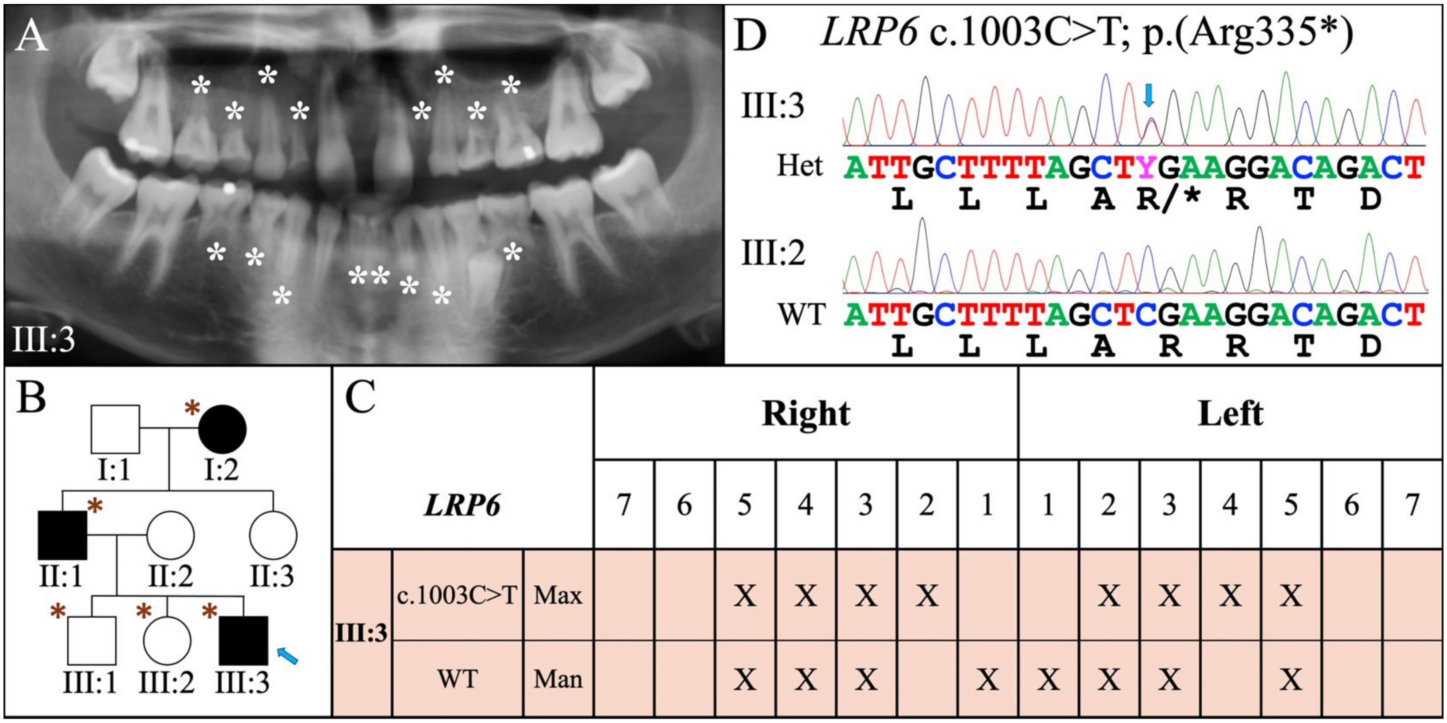

LRP6			Right							Left						
			7	6	5	4	3	2	1	1	2	3	4	5	6	7
**III:3**	c.1003C>T	Max			X	X	X	X			X	X	X	X		
	WT	Man			X	X	X		X	X	X	X		X		

Figure S9. Oligodontia Family 9 Heterozygous with the *LRP6* defect: c.1003C>T, p.(Arg335*). ***A:*** Panoramic radiograph of the proband (III:3) taken at age 12 years 2 months. A star marks the location of each absent (undeveloped) permanent tooth. ***B:*** Pedigree of Family 9. Asterisks mark the 5 subjects recruited in this study. Their genotypes are I:2 +/-, II:1 +/-, III:1+/+, III:2 +/+, and III:3 +/-. The mandibular first molars of III:3 show taurodontism. ***C:*** Summary chart showing the sites of tooth agenesis (X) for the proband (III:3), who lacked 16 permanent teeth and was includedin the data analysis. Subjects I:2 and II:1 were reported to have tooth agenesis, and were heterozygous for the same *LRP6* defect as the proband (not shown), but no radiographs were provided to ascertain the extent of the tooth agenesis. ***D:*** The chromatogram shows the heterozygous *LRP6* stop gain variant, and the wild-type reference sequence from subject III:2. Sequences altered by mutation are in magenta. Y = T or C. Subject III:1 was also wild-type (not shown) and affected subjects I:2 and II:1 were heterozygous for the *LRP6* defect (not shown), but we were unable to ascertain their numbers of teeth missing due to agenesis. The NCBI reference sequence designations for this *LRP6* variant are (NG_016168.2: g.90465C>T; NG_016168.2(LRP6_v001): c.1003C>T, p.(Arg335*).

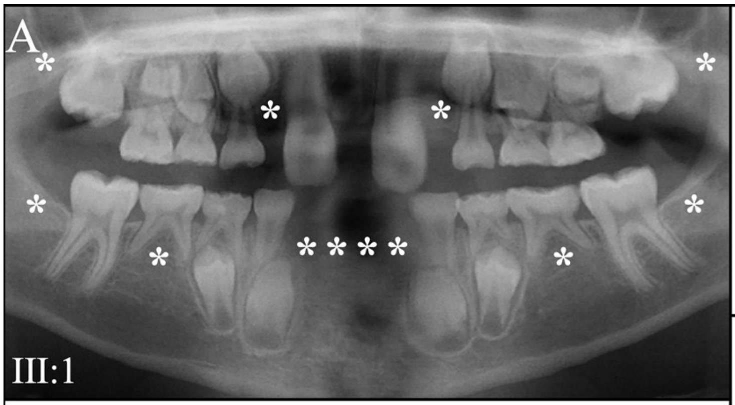

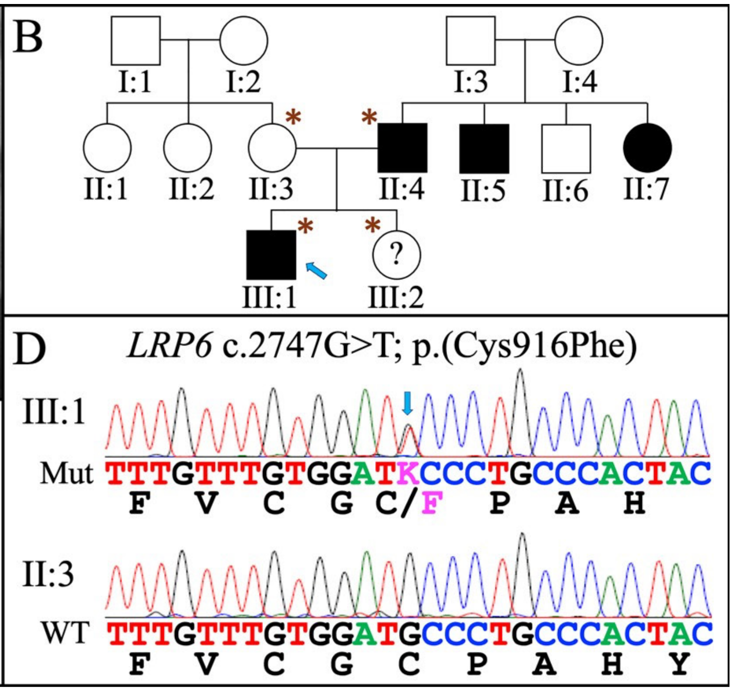

C

LRP6			Right							Left						
			7	6	5	4	3	2	1	1	2	3	4	5	6	7
**III:1**	c.2747G>T	Max	X					X			X					X
	WT	Man	X		X			X	X	X	X			X		X

Figure S10. Oligodontia Family 10 Heterozygous with the *LRP6* defect: c.2747G>T, p.(Cys916Phe). ***A:*** Panoramic radiograph of the proband (III:1) taken at age 10 years. A star marks the location of each absent (undeveloped) permanent tooth. ***B:*** Pedigree of Family 10. Asterisks mark the 4 subjects recruited in this study.Their genotypes are II:3 +/+, II:4 +/-, III:1+/-, and III:2 +/-. ***C:*** Summary chart showing the sites of tooth agenesis (X) for the proband (III:1), who lacked 12 permanent teeth and was included in the data analysis. Subject II:4 was reported to have tooth agenesis, but no radiographs were provided, and subject III:2 (at age 3) was too young to assess agenesis of permanent teeth. Both of these subjects were heterozygous for the same *LRP6* defect as the proband (not shown). ***D:*** The chromatograms show*LRP6* missense variant c.2747G>T, p.(Cys916Phe) in the proband (III:1) and reference sequence in subject II:3. Sequences altered by mutation are in magenta. K = T or G. The NCBI reference sequence designations for this *LRP6* variant are (NG_016168.2: g.113005G>T; NG_016168.2(LRP6_v001): c.2747G>T, p.(Cys916Phe).

Figure S11A Family 1 Chromatograms. *WNT10A* Exon 1: NG_012179.1:g.5562_5568dup; NM_025216.2:c.99_105dup; NP_079492.2:p.(Met36Cysfs*).

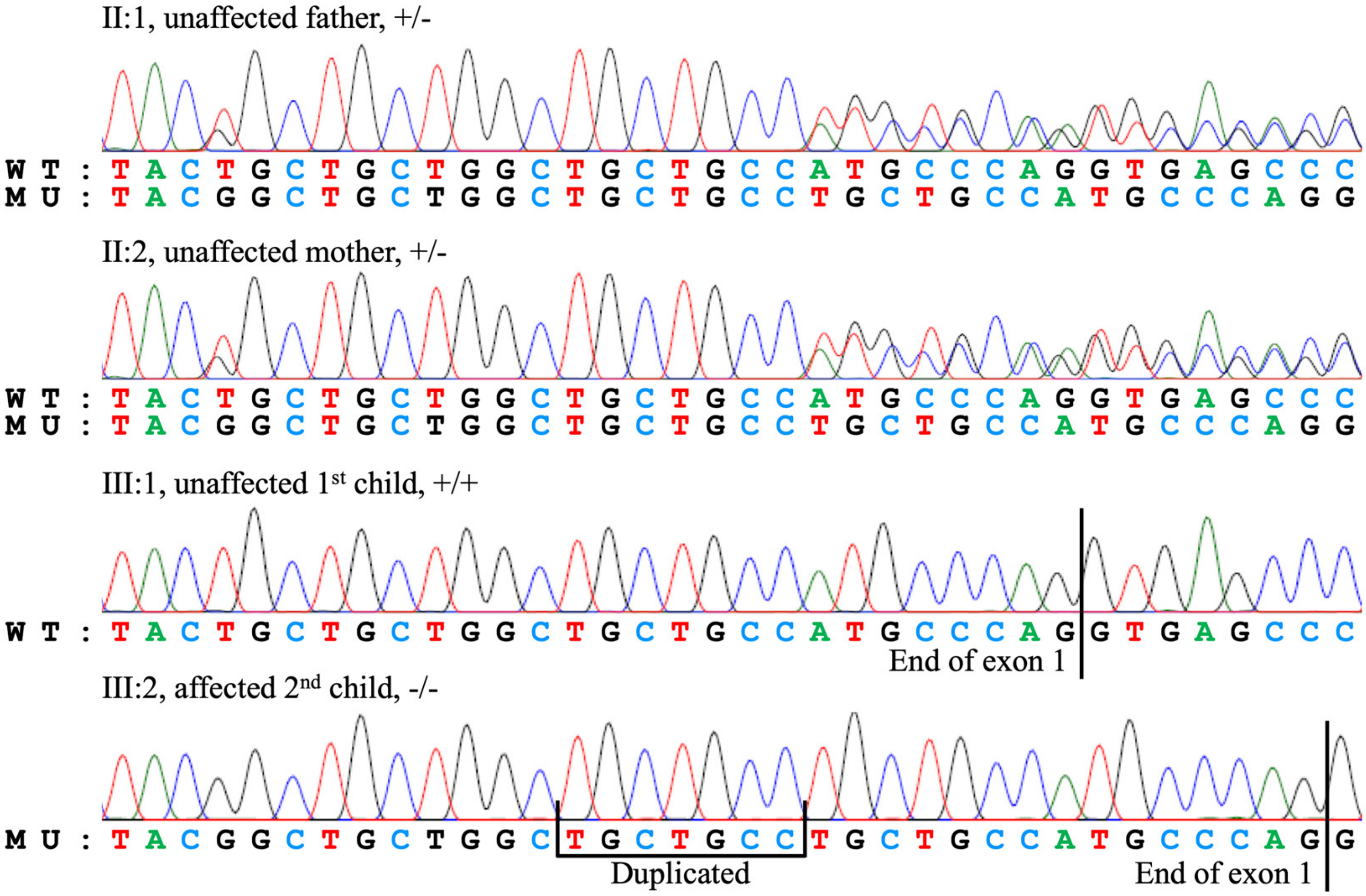

Figure S11B. Family 2 Chromatograms. *WNT10A* Exon 3: NG_012179.1:g.14508G>A; NM_025216.2:c.433G>A; NP_079492.2:p.(Val145Met)

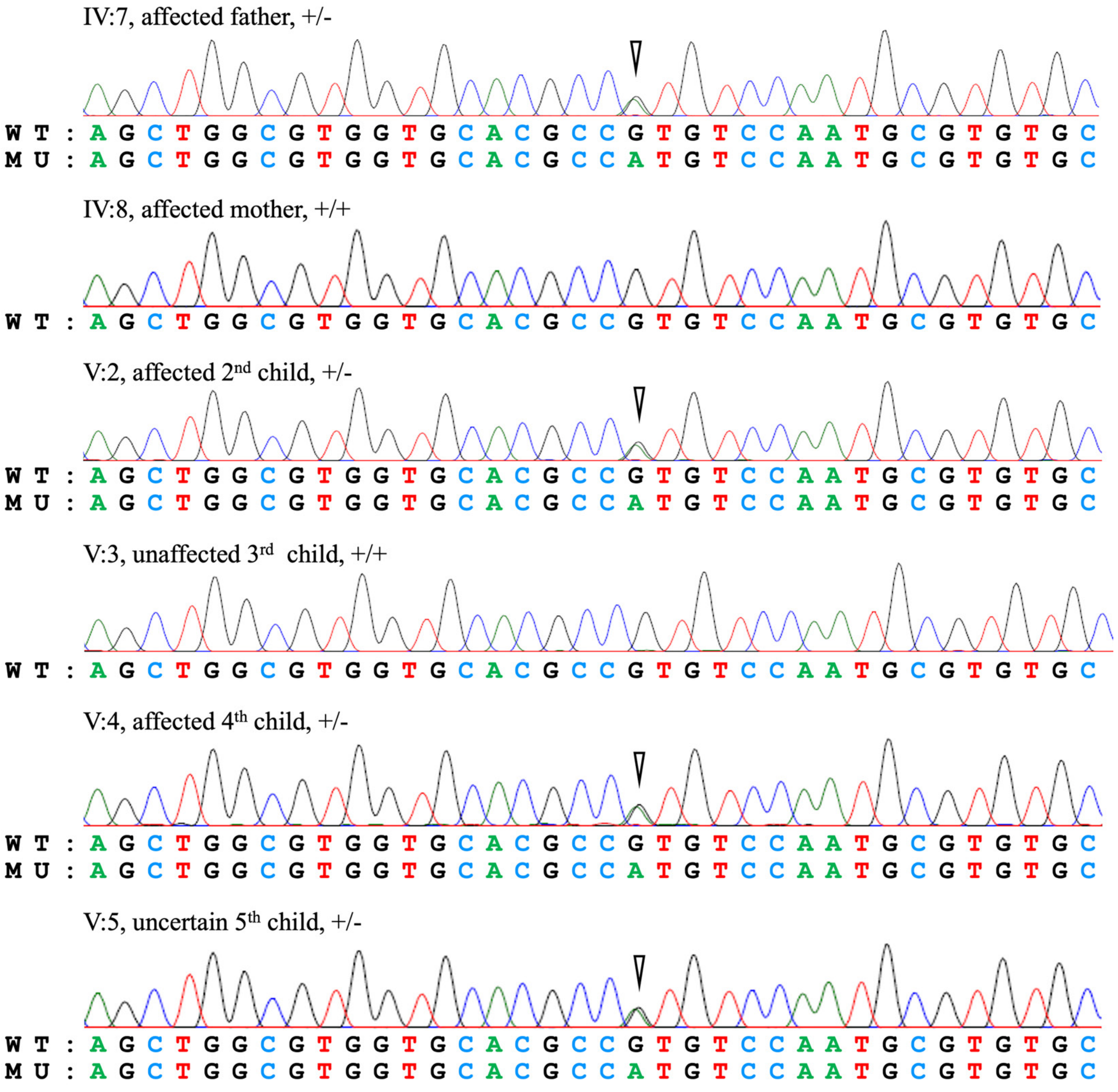

Figure S11B. Family 2 Chromatograms (continued). 2. *WNT10A* Exon 3: NG_012179.1:g.14757T>A; NM_025216.2:c.682T>A; NP_079492.2:p.(Phe228Ile)

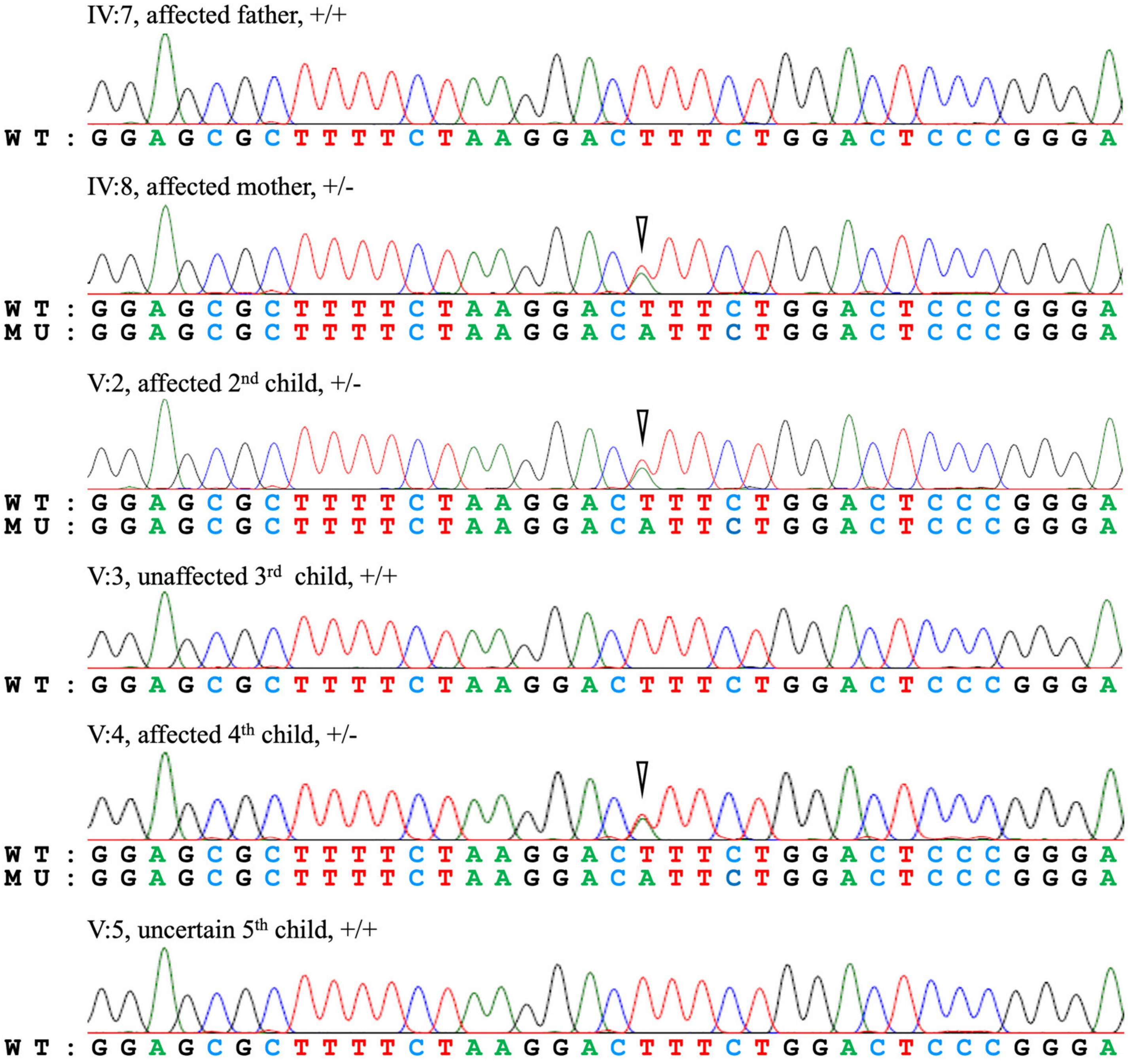

Figure S11C. Family 3 Chromatograms. *WNT10A* Exon 3: NG_012179.1:g.14508G>A; NM_025216.2:c.433G>A; NP_079492.2:p.(Val145Met)

III:9, affected father, +/-

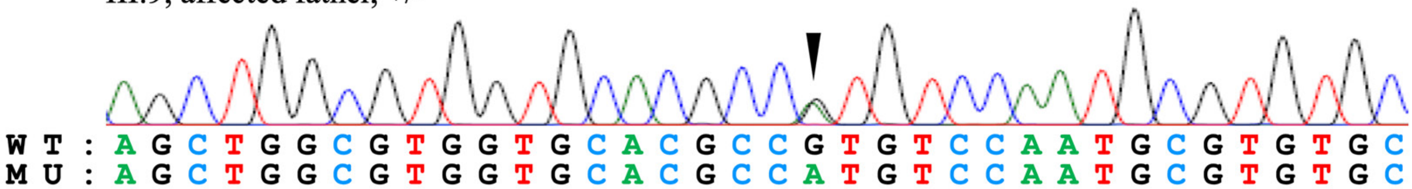

III:10, unaffected mother, +/-

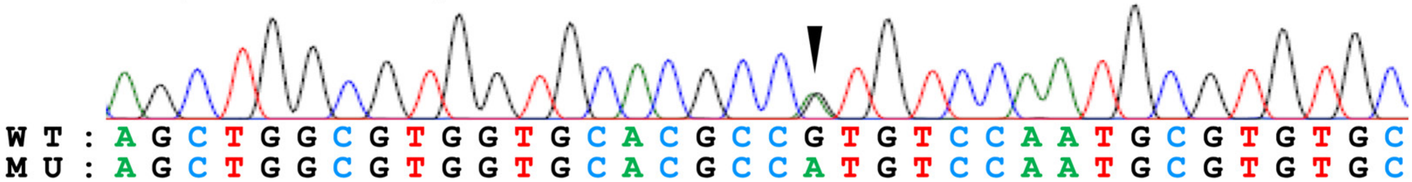

IV:1, affected 1st child, -/-

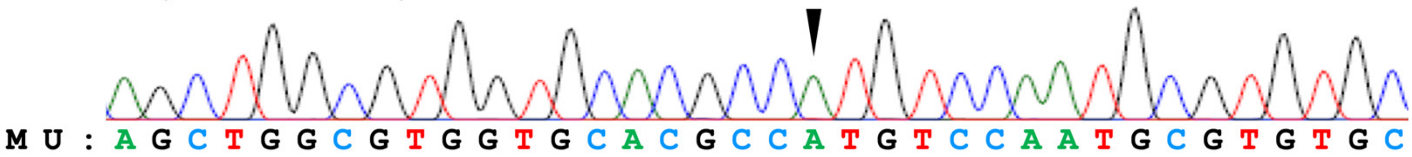

IV:2, uncertain 2nd child, +/-

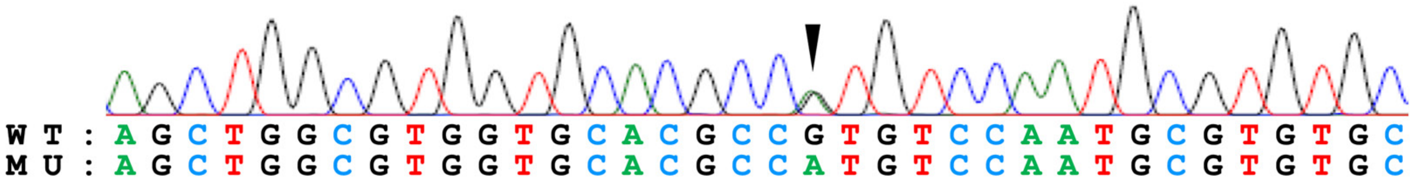

Figure S11D. Family 4 Chromatograms 1. *WNT10A* Exon 2: NG_012179.1:g.6833C>G; NM_025216.2:c.318C>G; NP_079492.2:p.(Asn106Lys)

II:4, affected mother, +/-

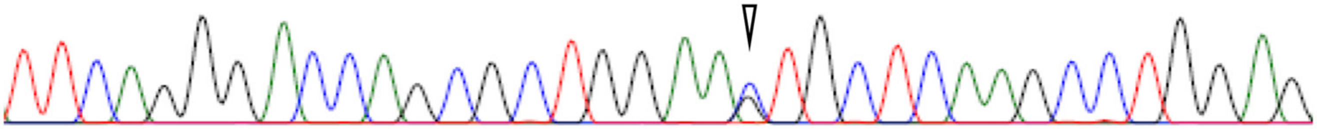

WT: TTCAGGGACCAGCGCTGGAACTGCTCAAGCCTGGAG
MU: TTCAGGGACCAGCGCTGGAAGTGCTCAAGCCTGGAG

III:2, unaffected elder sister, +/+

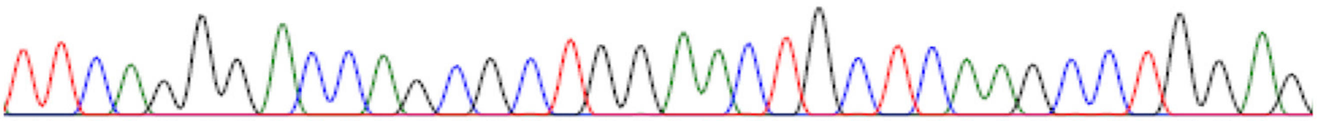

WT: TTCAGGGACCAGCGCTGGAACTGCTCAAGCCTGGAG

III:3, affected younger sister, +/-

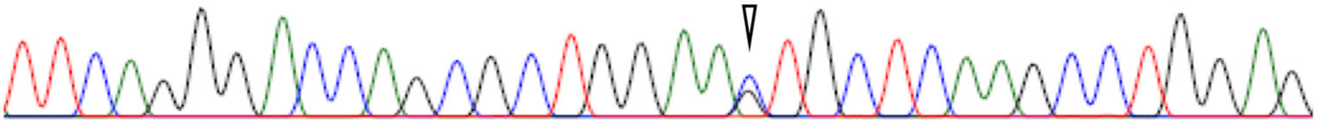

WT: TTCAGGGACCAGCGCTGGAACTGCTCAAGCCTGGAG
MU: TTCAGGGACCAGCGCTGGAAGTGCTCAAGCCTGGAG

Figure S11D. Family 4 chromatograms 2. *WNT10A* Exon 3: NG_012179.1:g.14757T>A; NM_025216.2:c.682T>A; NP_079492.2:p.(Phe228Ile)

II:4, affected mother, +/+

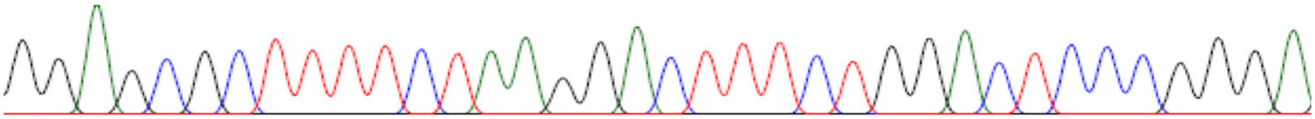

WT: GGAGCGCTTTTCTAAGGACTTTCTGGACTCCCGGGA

III:2, unaffected elder sister, +/+

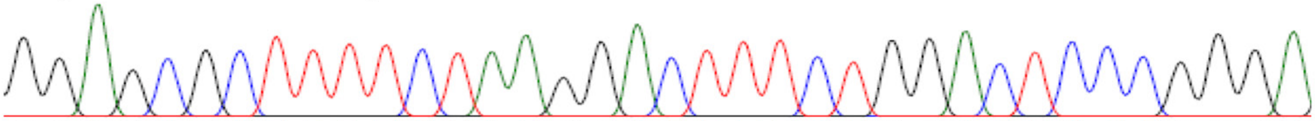

WT: GGAGCGCTTTTCTAAGGACTTTCTGGACTCCCGGGA

III:3, affected younger sister, +/-

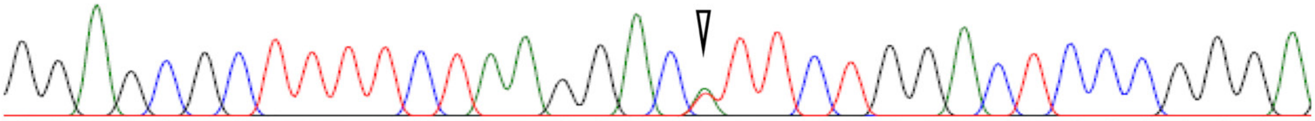

WT: GGAGCGCTTTTCTAAGGACTTTCTGGACTCCCGGGA
MU: GGAGCGCTTTTCTAAGGACATTCTGGACTCCCGGGA

Figure S11F. Family 6 Chromatograms. *WNT10A* Exon 2: NG_012179.1:g.6836C>A; NM_025216.2:c.321C>A; NP_079492.2:p.(Cys107*)

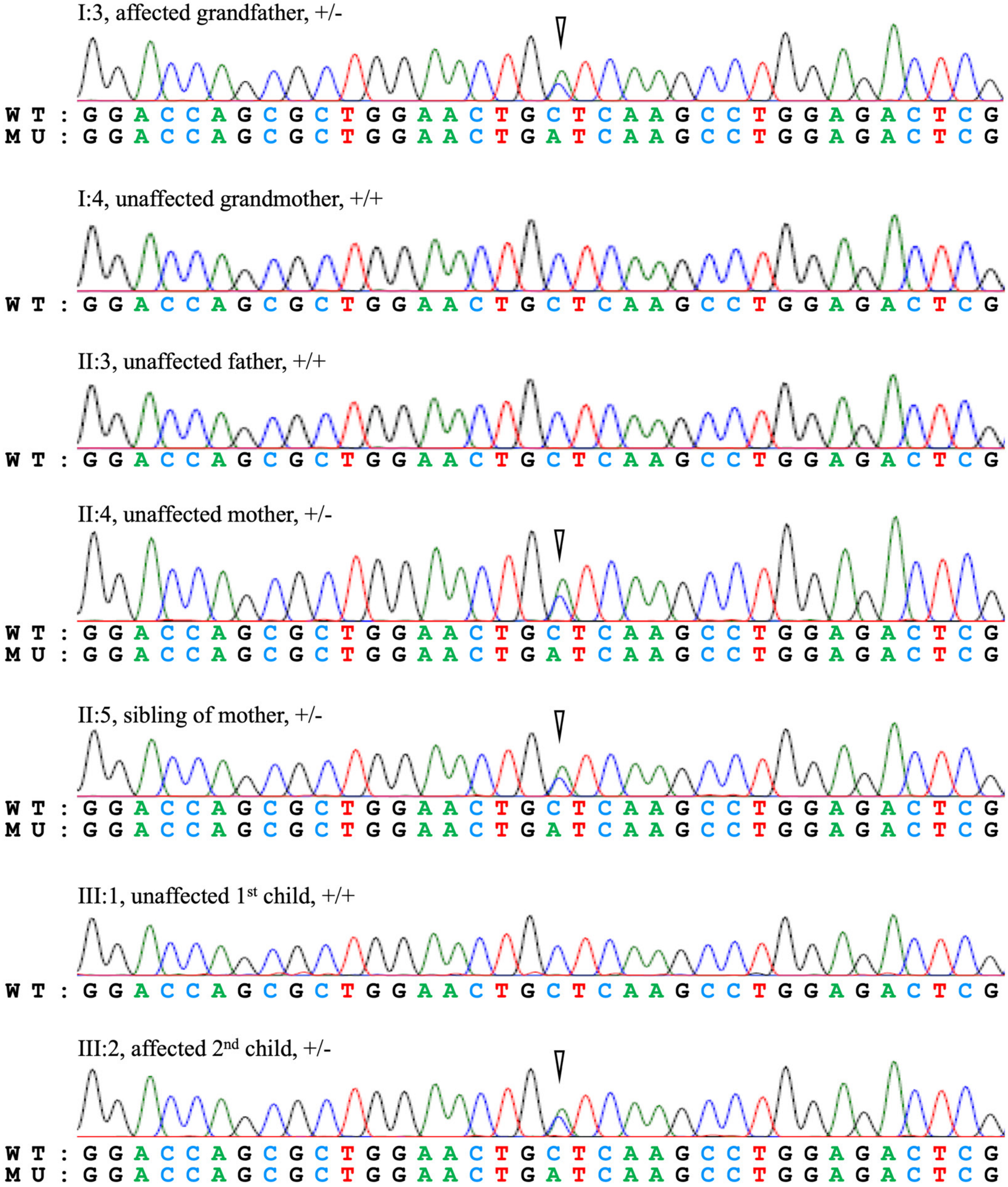

Figure S11G. Family 7 Chromatograms. *WNT10A* Exon 3: NG_012179.1:g.14757T>A; NM_025216.2:c.682T>A; NP_079492.2:p.(Phe228Ile)

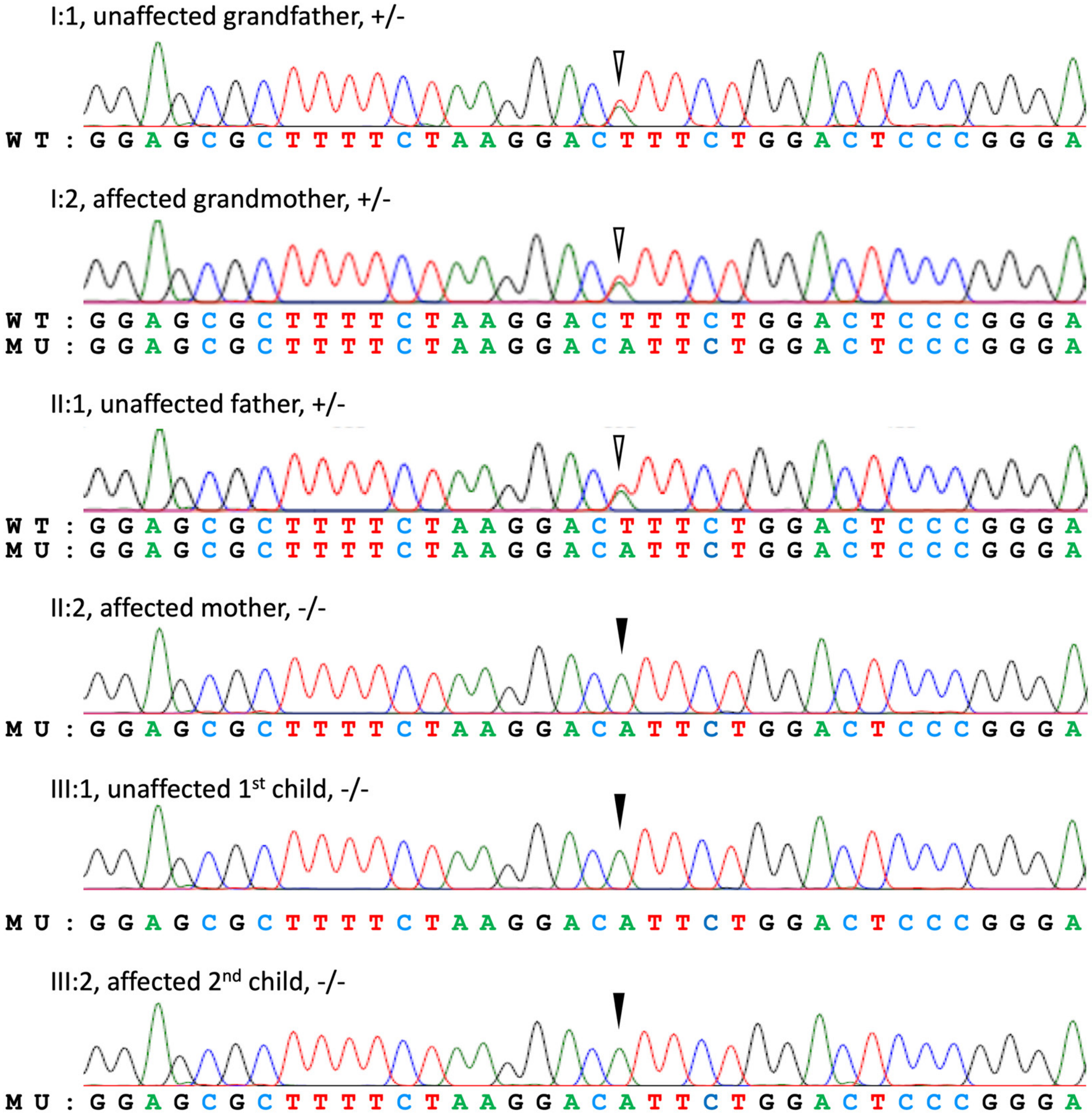

Figure S11H. Family 8 Chromatograms. *EDAR* Exon 7: NG_008257.1:g.83352C>T; NM_022336.3:c.581C>T; NP_071731.1:p.(Thr194Ile)

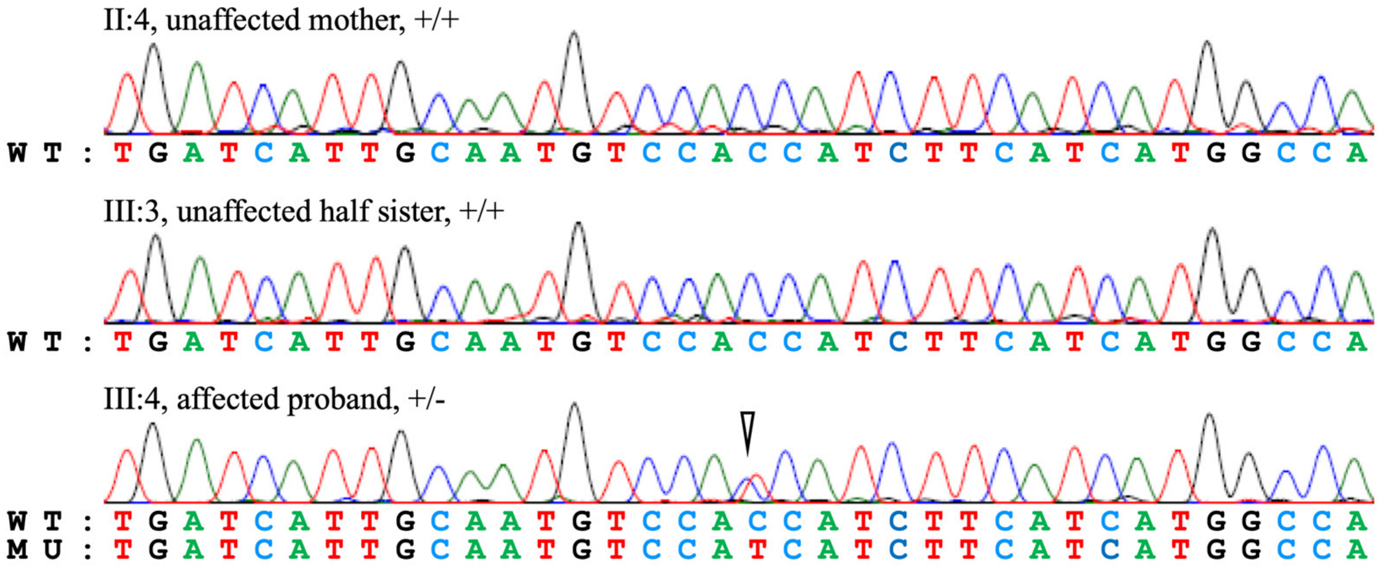

Figure S11i. Family 9 Chromatograms. *LRP6* Exon 6: NG_016168.1:g.90465C>T; NM_002336.2:c.1003C>T; NP_002327.2:p.(Arg335*)

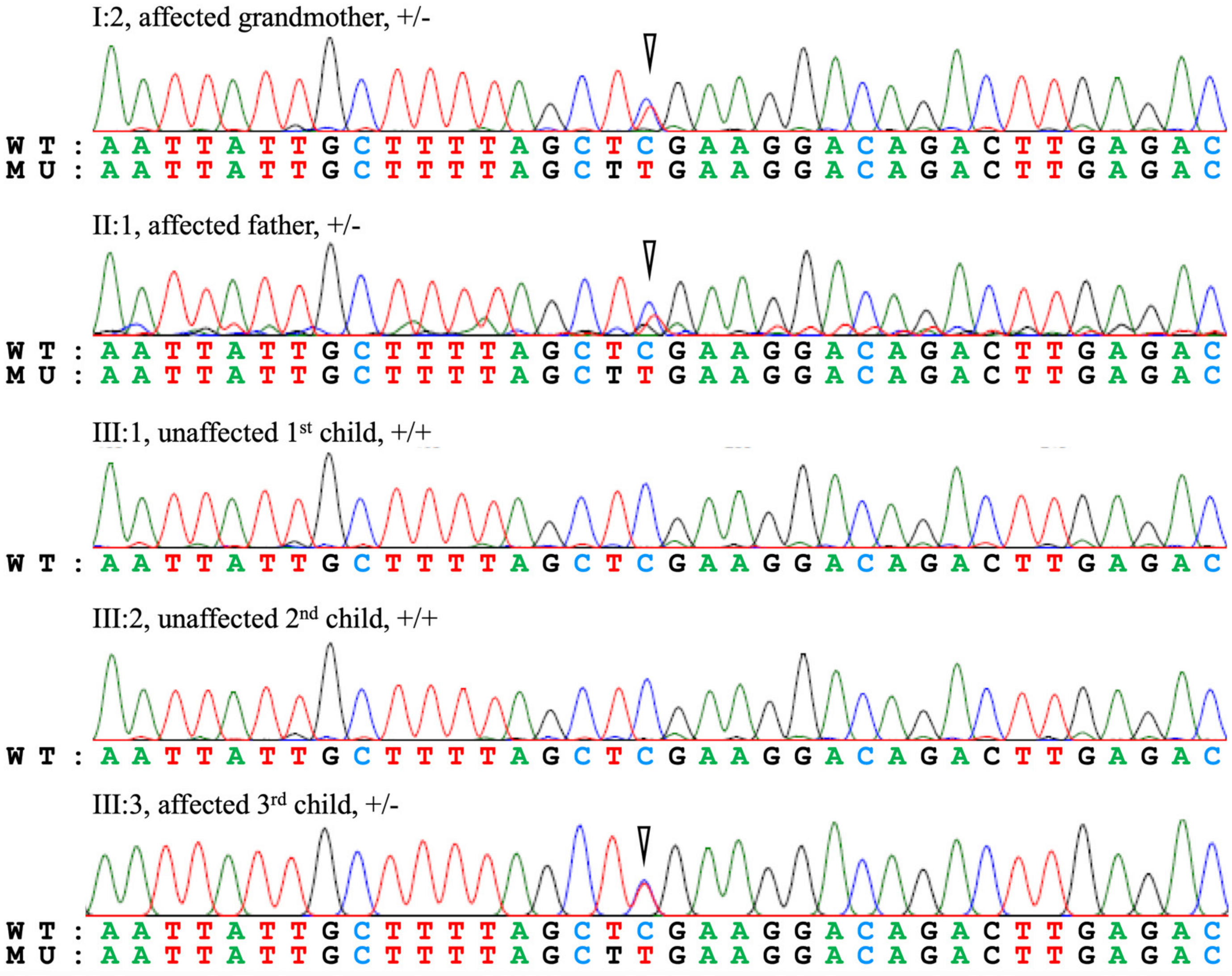

Figure S11J. Family 10 Chromatograms. *LRP6* Exon 12: NG_016168.1:g.113005G>T; NM_002336.2:c.2747G>T; NP_002327.2:p.(Cys916Phe)

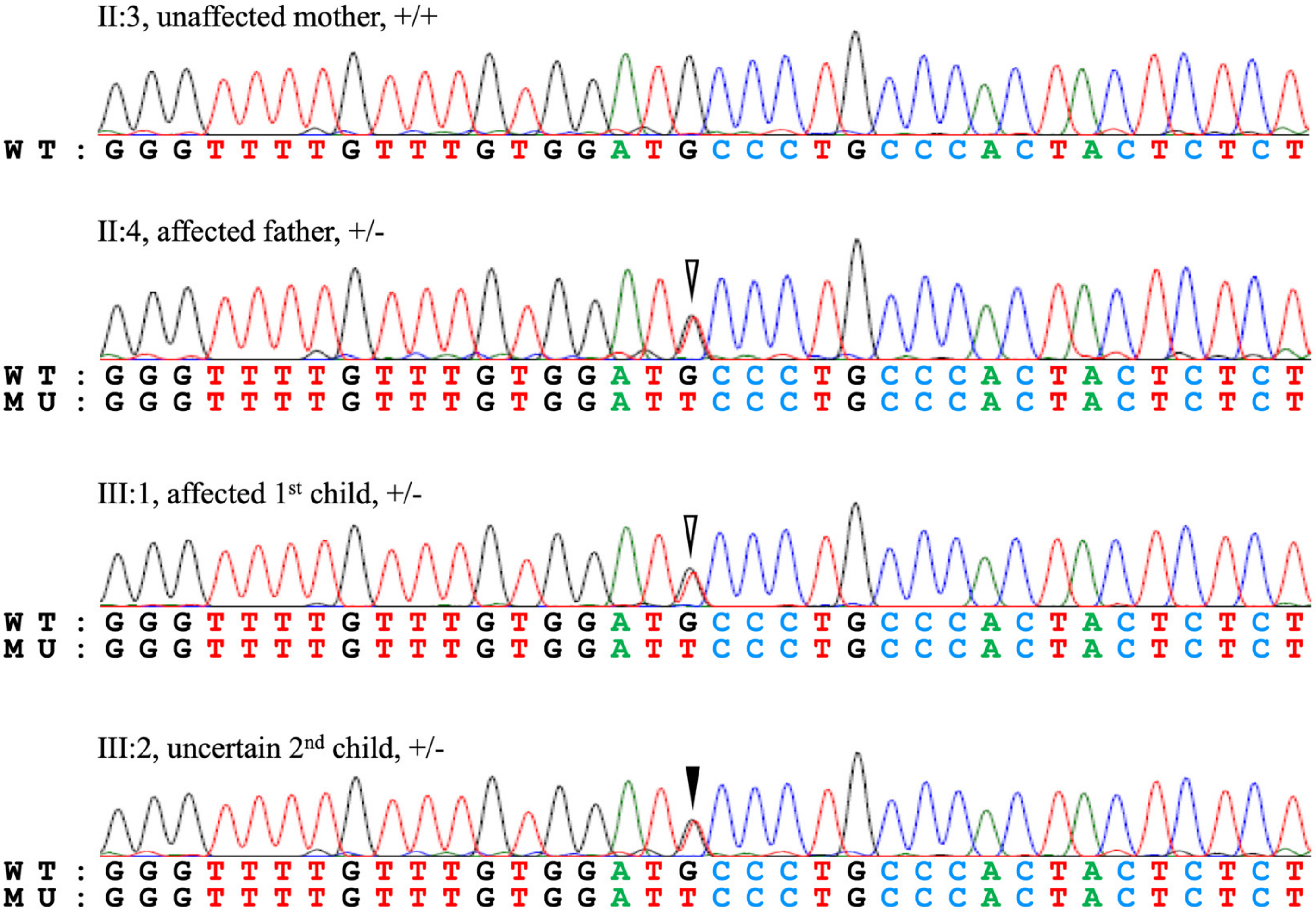

Table S1. List of articles reviewed in this study with brief summaries of their specific findings.

					MAX							MAN								
Article	Year	Patients' region	Gene name	Number of cases	Mo2	Mo1	PM2	PM1	Ca	LI	CI	Mo2	Mo1	PM2	PM1	Ca	LI	CI	syndromic phenotype	
Functional analysis of a novel missense mutation in AXIN2 associated with non-syndromic tooth agenesis	2016	China	AXIN2	1	0	0	2	2	0	0	0	0	0	2	1	0	0	2	/	
Axis inhibition protein 2 polymorphisms may be a risk factor for families with isolated oligodontia	2014	China	AXIN2	2	2	0	4	3	4	3	0	2	0	4	2	0	2	2	/	
A Novel AXIN2 Missense Mutation Is Associated with Non-Syndromic Oligodontia	2015	China	AXIN2	1	0	0	2	2	0	0	0	0	0	2	1	0	0	0	/	
Novel missense mutations in the AXIN2 gene associated with non-syndromic oligodontia	2014	China	AXIN2	2	0	0	4	1	1	4	2	0	0	4	2	1	3	2	/	
Isolated Oligodontia Associated With Mutations in EDARADD, AXIN2, MSX1, and PAX9 Genes	2011	Sweden	AXIN2	3	1	2	6	3	0	2	0	2	2	3	0	0	0	4	/	
AXIN2-Associated Autosomal Dominant Ectodermal Dysplasia and Neoplastic Syndrome	2011	USA	AXIN2	1	2	0	0	0	2	0	0	2	0	0	0	1	2	2	sparse eyebrows, slightly upslanting palpebral fissures, and thin upper vermillion;multiple fundic gland	Ectodermal Dysplasia and Neoplastic Syndrome
Phenotypic confirmation of oligodontia, colorectal polyposis and cancer in a family carrying an exon 7 nonsense variant in the AXIN2 gene	2019	Australia	AXIN2	2	4	2	2	2	2	2	2	0	3	1	3	0	0	2	/	
Mutations in AXIN2 Cause Familial Tooth Agenesis and Predispose to Colorectal Cancer	2004	Finnish	AXIN2	7	12	8	10	9	4	9	0	11	10	14	6	4	9	11	colorectal neoplasia	
Mutations in AXIN2 Cause Familial Tooth Agenesis and Predispose to Colorectal Cancer	2004	Finnish	AXIN2	4	5	4	8	5	2	8	0	3	3	8	2	3	7	7	/	
Targeted next-generation sequencing (NGS) analysis of mutations in nonsyndromic tooth agenesis candidate genes	2021	Turkey	AXIN2	1	0	0	2	0	0	2	0	0	0	2	0	0	0	0	/	
A new Hypo/Oligodontia Syndrome: Carvajal/naxos Syndrome Secondary to Desmoplakin-dominant Mutations	2011	France	DSP	1	2	0	2	0	2	1	0	2	0	2	0	0	0	0	woolly hair, palmoplantar keratoderma, and dilated cardiomyopathy	Carvajal/Naxos syndrome
Deleterious Variants in WNT10A, EDAR, and EDA Causing Isolated and Syndromic Tooth Agenesis: A Structural Perspective from MolecularDynamics Simulations	2019	Pakistan, Egypt, Saudi Arabia, and Syria	EDA	1	0	0	0	0	0	2	0	0	0	2	0	0	2	2	/	
Whole Genome Sequencing Reveals Novel Non- Synonymous Mutation in Ectodysplasin A (EDA) Associated with Non-Syndromic X-Linked Dominant Congenital Tooth Agenesis	2014	Indian	EDA	3	2	0	2	2	1	0	4	0	0	3	2	5	6	6	/	
Oligodontia and curly Hair Occur with Ectodysplasin-A Mutations	2014	korea	EDA	3	1	0	6	2	6	6	2	3	2	6	4	2	6	4	/	
Non-syndromic Tooth Agenesis Associated with a Nonsense Mutation in Ectodysplasin-A (EDA)	2013	Sweden	EDA	2	0	0	0	0	3	3	0	0	0	1	0	0	4	4	/	
Candidate Gene Analysis of Tooth Agenesis Identifies Novel Mutations in Six Genes and Suggests Significant Role for WNT and EDA Signaling and Allele Combinations	2013	Finland	EDA	3	0	0	2	5	2	6	0	0	0	1	2	0	6	4	/	
EDA Gene Mutations Underlie Non-syndromic Oligodontia	2009	China	EDA	4	2	0	6	6	6	8	1	1	0	6	6	3	6	5	/	
Novel mutations identified in patients with tooth agenesis by whole - exome sequencing	2019	China	EDA	1	2	2	2	2	2	2	2	2	2	2	2	2	2	2	thin or wrinkled skin sparse or curly hair Hypohidrosis	Ectodermal Dysplasia
Novel missense mutation in the EDA gene in a family affected by oligodontia	2016	Germany	EDA	2	0	0	0	2	2	4	0	0	0	1	1	0	4	4	one patient presents sparse, wiry hair.The other one presents an abnormal ear shape and sparse, wiry hair	Ectodermal Dysplasia
Missense mutation of the EDA gene in a Jordanian family with X-linked hypohidrotic ectodermal dysplasia: phenotypic appearance and speech problems	2010	Jordanian	EDA	1	2	2	0	0	0	2	0	2	1	2	2	2	0	2	heat intolerance, sparse hair (hypotrichosis);speech problems, and damaged eccrine glands, resulting in reduced sweating (anhidrosis)	HED (Hypohidrotic Ectodermal Dysplasia)
A novel EDAR missense mutation identified by whole-exome sequencing with non-syndromic tooth agenesis in a Chinese family	2021	China	EDA	3	0	0	4	0	1	6	0	0	0	6	1	0	6	6	/	
Comparative analysis of rare EDAR mutations and tooth agenesis pattern in EDAR - and EDA - associated nonsyndromic oligodontia	2020	China	EDAR	8	5	4	9	12	8	7	0	4	2	11	5	4	9	6	/	
Novel EDAR mutation in tooth agenesis and variable associated features	2020	Turkey	EDAR	2	0	0	0	0	0	4	0	0	1	0	1	3	4	1	/	
Candidate Gene Analysis of Tooth Agenesis Identifies Novel Mutations in Six Genes and Suggests Significant Role for WNT and EDA Signaling and Allele Combinations	2013	Finland	EDAR	3	0	0	4	1	4	6	0	0	0	4	2	0	4	0	/	
A novel EDAR missense mutation identified by whole-exome sequencing with non-syndromic tooth agenesis in a Chinese family	2021	China	EDAR	1	2	2	2	0	1	1	2	2	1	2	0	0	1	2	/	
Candidate Gene Analysis of Tooth Agenesis Identifies Novel Mutations in Six Genes and Suggests Significant Role for WNT and EDA Signaling and Allele Combinations	2013	Finland	EDARADD	4	0	0	6	4	3	4	0	3	0	8	5	0	0	0	/	
Isolated Oligodontia Associated With Mutations in EDARADD, AXIN2, MSX1, and PAX9 Genes	2011	Sweden	EDARADD	1	0	0	2	0	0	0	0	2	0	2	0	0	0	0	/	
Whole-Exome Sequencing Identifies Novel Variants for Tooth Agenesis	2018	Turkey	KREMEN1	1	2	0	0	2	0	2	0	1	0	1	1	1	2	2	/	
Whole-Exome Sequencing Identifies Novel Variants for Tooth Agenesis	2018	Turkey	KREMEN1	2	4	0	2	4	2	4	0	3	0	3	3	3	4	4	mild clinical findings of ectodermal dysplasia (sparse hair, dry skin, sparse eyebrows and eyelashes, pro_x005f_x0002_truded lips, and heat intolerance)	Ectodermal Dysplasia
Mutation of KREMEN1, a modulator of Wnt signaling, is responsible for ectodermal dysplasia including oligodontia in Palestinian families	2016	Palestine	KREMEN1	9	2	0	0	2	2	18	2	2	0	2	2	16	18	18	facial features include abnormal hair distribution of the scalp, low hairline with forehead fuzziness, broad and low nose bridge, columella extending with age, thick lips, slight ocular hypertelorism, and downward slanting of the palpebral fissures.	Ectodermal Dysplasia
Mutation of KREMEN1, a modulator of Wnt signaling, is responsible for ectodermal dysplasia including oligodontia in Palestinian families	2016	Palestine	KREMEN1	3	0	0	0	0	0	8	0	0	0	0	0	0	8	8	/	/
Lrp6 Dynamic Expression in Tooth Development and Mutations in Oligodontia	2020	China	LRP6	4	4	0	7	5	7	6	0	6	1	6	1	1	3	2	/	
Whole-Exome Sequencing Identifies Novel Variants for Tooth Agenesis	2018	Turkish	LRP6	1	0	0	2	2	0	2	0	0	0	2	2	2	2	2	/	
Loss-of-Function Mutations in the WNT Co-receptor LRP6 Cause Autosomal-Dominant Oligodontia	2015	Netherland	LRP6	4	3	0	7	6	6	8	0	5	0	8	4	4	7	7	/	
Lrp6 Dynamic Expression in Tooth Development and Mutations in Oligodontia	2020	China	LRP6	1	2	2	2	2	0	2	0	0	0	2	2	0	2	2	sparse hair and hypohidrosis	Ectodermal Dysplasia
Concurrent manifestation of oligodontia and thrombocytopenia caused by a contiguous gene deletion in 12p13.2: A three- generation clinical report	2019	Netherlands	LRP6	3	0	0	0	0	6	6	0	0	0	0	0	5	5	6	/	
A novel missense mutation of LRP6 identified by whole-exome sequencing in a Chinese family with non-syndromic tooth agenesis	2021	China	LRP6	1	2	0	2	2	0	2	0	2	0	2	2	0	1	2	/	
Two novel mutations in MSX1 causing oligodontia	2020	China	MSX1	2	4	0	3	4	2	2	2	4	0	2	2	1	2	2	/	
A novel mutation of MSX1 inherited from maternal mosaicism causes a severely affected child with nonsyndromic oligodontia	2020	China	MSX1	1	0	0	2	2	0	0	0	1	2	2	0	2	0	2	/	
A novel mutation of MSX1 in oligodontia inhibits odontogenesis of dental pulp stem cells via the ERK pathway	2018	China	MSX1	5	0	0	12	10	0	1	0	0	2	10	0	0	0	0	/	
Next generation sequencing reveals a novel nonsense mutation in MSX1 gene related to oligodontia	2018	GERMANY	MSX1	2	2	0	4	4	0	4	0	2	0	4	0	0	0	2	/	
A novel MSX1 intronic mutation associated with autosomal dominant non- syndromic oligodontia in a large Chinese family pedigree	2016	China	MSX1	7	9	4	5	10	4	1	3	11	6	7	7	3	4	4	/	
Mutations in MSX1, PAX9 and MMP20 genes in Saudi Arabian patients with tooth agenesis	2016	Saudi Arabian	MSX1	2	0	0	4	4	0	0	0	0	0	4	2	0	0	0	/	
An Aberrant Splice Acceptor Site Due to a Novel Intronic Nucleotide Substitution in MSX1 Gene Is the Cause of Congenital Tooth Agenesis in a Japanese Family	2015	Janpan	MSX1	3	2	0	6	1	1	1	1	4	3	4	1	1	1	3	/	
Characterization of Novel MSX1 Mutations Identified in Japanese Patients with Nonsyndromic Tooth Agenesis	2014	Japan	MSX1	3	1	2	4	4	1	2	2	0	2	6	6	2	1	2	/	
A novel non-stop mutation in MSX1 causing autosomal dominant non-syndromic oligodontia	2014	China	MSX1	1	0	0	2	2	1	2	1	2	0	2	0	1	1	2	/	
Novel nonsense mutation in MSX1 in familial nonsyndromic oligodontia: subcellular localization and role of homeodomain/MH4	2014	Japan	MSX1	2	2	0	4	4	0	0	0	2	2	4	0	0	0	4	/	
Clinical and genetic evaluation of a Chinese family with isolated oligodontia	2013	China	MSX1	2	0	0	4	3	1	4	0	0	0	4	3	0	2	4	/	
Candidate Gene Analysis of Tooth Agenesis Identifies Novel Mutations in Six Genes and Suggests Significant Role for WNT and EDA Signaling and Allele Combinations	2013	Finland	MSX1	2	0	0	4	4	0	1	0	2	0	4	0	0	0	4	/	
Isolated Oligodontia Associated With Mutations in EDARADD, AXIN2, MSX1, and PAX9 Genes	2011	Sweden	MSX1	2	2	2	4	3	0	3	0	2	2	4	0	0	1	2	/	
Mutations in the Human Homeobox MSX1 Gene in the Congenital Lack of Permanent Teeth	2009	Poland	MSX1	2	0	0	2	0	0	2	0	0	0	4	2	0	0	2	/	
Identification of a novel missense mutation of MSX1 gene in Chinese family with autosomal-dominant oligodontia	2008	Chinese	MSX1	2	2	0	1	0	3	4	2	0	0	4	0	2	2	2	/	
A novel c.581C>T transition localized in a highly conserved homeobox sequence of MSX1: is it responsible for oligodontia?	2006	Poland	MSX1	1	0	0	1	2	0	0	0	2	0	2	1	0	0	2	/	
Novel MSX1 Frameshift Causes Autosomal-dominant Oligodontia	2006	Korea	MSX1	2	0	0	4	2	0	2	0	2	0	4	0	0	4	0	/	
A novel missense mutation in MSX1 underlies autosomal recessive oligodontia with associated dental anomalies in Pakistani families	2006	Pakistani	MSX1	2	2	2	2	2	0	2	1	4	4	4	2	0	3	2	/	
MSX1 Gene is Deleted in Wolf-Hirschhorn Syndrome Patients with Oligodontia	2003	Finish	MSX1	5	5	4	10	9	0	5	0	5	10	10	3	3	2	6	mental and growth retardation, seizures, and hypospadia	Wolf-Hirschhorn syndrome
Targeted next-generation sequencing (NGS) analysis of mutations in nonsyndromic tooth agenesis candidate genes	2021	Turkey	MSX1	1	0	0	2	2	0	2	0	0	0	2	0	0	0	2	/	
Novel MSX1 variants identified in families with nonsyndromic oligodontia	2021	China	MSX1	8	4	2	16	14	2	7	6	10	5	14	5	1	1	11	/	

(Continued on next page)

Functional study of novel PAX9 variants: The paired domain and non-syndromic oligodontia	2020	China	PAX9	3	6	4	2	5	4	3	2	6	6	5	0	0	0	4	/	
Familial oligodontia and regional odontodysplasia associated with a PAX9 initiation codon mutation	2019	Finland	PAX9	4	7	6	6	4	2	4	1	8	4	4	2	0	0	4	/	
Nine Novel PAX9 Mutations and a Distinct Tooth Agenesis Genotype-Phenotype	2018	China	PAX9	10	19	20	16	9	5	7	2	19	9	8	5	4	6	11	/	/
A novel G to A transition at initiation codon and exon-intron boundary of PAX9 identified in association with familial isolated oligodontia	2017	Indian	PAX9	6	12	8	9	1	5	2	4	11	9	3	0	0	0	5		/
Characterization of PAX9 variant P20L identified in a Japanese family with tooth agenesis	2017	Janpan	PAX9	2	4	4	3	0	0	0	0	4	0	0	0	0	0	0	/	
A novel PAX9 mutation causing oligodontia	2017	Malta	PAX9	1	2	2	0	0	0	0	0	2	2	0	0	0	0	2	/	
Mutations in WNT10B Are Identified in Individuals with Oligodontia	2016	China	PAX9	2	4	4	2	2	2	2	0	4	2	2	0	0	0	2	/	
A novel initiation codon mutation of PAX9 in a family with oligodontia	2015	China	PAX9	5	10	8	6	0	3	2	4	10	7	0	0	1	1	5	/	
A Nonsyndromic Autosomal Dominant Oligodontia with A Novel Mutation of Pax9-A Clinical and Genetic Report	2015	Indian	PAX9	2	2	0	2	0	3	4	0	2	0	3	0	4	2	2	/	
A screen of a large Czech cohort of oligodontia patients implicates a novel	2015	Czech	PAX9	3	6	6	6	0	0	4	0	6	1	2	0	0	0	1	/	
Novel PAX9 mutations cause non-syndromic tooth agenesis	2014	Japan	PAX9	2	4	4	2	0	0	0	0	4	0	0	0	0	0	2	/	
Novel missense mutation in PAX9 gene associated with familial tooth agenesis	2012	Brazil	PAX9	3	1	0	4	5	2	0	0	0	0	5	4	0	0	1	/	
Candidate Gene Analysis of Tooth Agenesis Identifies Novel Mutations in Six Genes and Suggests Significant Role for WNT and EDA Signaling and Allele Combinations	2013	Finland	PAX9	2	4	2	2	0	0	0	0	2	0	2	0	0	0	2	/	/
Novel missense mutations in PAX9 causing oligodontia	2012	China	PAX9	4	8	8	2	0	7	1	0	7	3	0	0	2	0	1		/
Sequence analysis of PAX9, MSX1 and AXIN2 genes in a Chinese oligodontia family	2011	China	PAX9	1	0	0	0	0	2	2	0	0	0	0	0	2	2	2	/	
Isolated Oligodontia Associated With Mutations in EDARADD, AXIN2, MSX1, and PAX9 Genes	2011	Sweden	PAX9	4	6	4	6	4	2	1	0	6	2	5	2	0	0	1		/
Mutations in the PAX9 gene in sporadic oligodontia	2010	Poland	PAX9	6	7	4	7	7	6	4	0	9	2	7	7	4	6	6	/	
Identification and Functional Analysis of Two Novel PAX9 Mutations	2009	China	PAX9	1	2	2	0	2	0	0	0	2	1	0	0	0	0	0	/	
Identification of a novel mutation in the PAX9 gene in a family affected by oligodontia and other dental anomalies	2007	Spain	PAX9	4	8	8	5	2	0	0	0	8	2	2	0	0	0	0	/	
A novel nonsense mutation in PAX9 is associated with marked variability in number of missing teeth	2007	Denmark	PAX9	3	6	4	5	1	1	0	0	6	0	4	0	0	2	3	/	
A novel mutation in PAX9 causes familial form of molar oligodontia	2006	Poland	PAX9	5	10	10	10	0	0	0	0	10	10	10	0	0	0	10	/	
Molecular characterization of a novel PAX9 missense mutation causing posterior tooth agenesis	2006	USA	PAX9	2	4	4	4	0	0	0	0	3	4	4	0	0	0	0	/	
Novel Mutation of the Initiation Codon of PAX9 Causes Oligodontia	2005	Chinese	PAX9	2	4	4	4	2	2	0	0	4	4	4	0	0	0	2	/	
A novel missense mutation in the paired domain of PAX9 causes non-syndromic oligodontia.	2004	USA	PAX9	1	2	2	2	0	2	0	0	2	1	0	0	0	0	2	/	
Novel mutation in the paired box sequence of PAX9 gene in a sporadic form of oligodontia	2003	Poland	PAX9	1	0	0	2	0	1	2	0	0	0	2	0	0	1	1	/	
A missense mutation in PAX9 in a family with distinct phenotype of oligodontia	2003	Finland	PAX9	4	8	5	6	0	3	4	0	8	0	6	0	1	0	4	/	
Mutational Analysis of Families Affected with Molar Oligodontia	2002	USA	PAX9	8	10	4	14	14	4	2	0	14	10	14	6	0	0	0	/	
Identification of a nonsense mutation in the PAX9 gene in molar oligodontia	2001	Finland	PAX9	5	10	7	8	4	4	10	0	9	6	5	2	4	3	5	/	
Novel PAX9 and COL1A2 Missense Mutations Causing Tooth Agenesis and OI/DGI without Skeletal Abnormalities	2012	European	PAX9	1	2	0	2	1	0	0	0	2	0	1	0	0	0	2	/	
Intragenic duplication--a novel causative mechanism for SATB2-associated syndrome	2014	Sweden	SATB2	1	0	0	2	0	2	0	0	0	0	2	0	2	2	0	intellectual disability, speech and language impairment, cleft palate, malformed teeth, and oligodontia.	SATB2-Associated Syndrome : moderate-severe intellectual disability (ID)
Recessive oligodontia linked to a homozygous loss-of- function mutation in the SMOC2 gene	2013	Pakistan	SMOC2	3	0	0	6	1	4	2	0	0	0	6	6	6	6	0	/	
Homozygosity Mapping and Candidate Prioritization Identify Mutations, Missed by Whole-Exome Sequencing, in SMOC2, Causing Major Dental Developmental Defects	2011		SMOC2	2	0	0	4	0	0	0	0	0	0	4	3	4	2	0	/	
Targeted next-generation sequencing (NGS) analysis of mutations in nonsyndromic tooth agenesis candidate genes	2021	Turkey	SMOC2	1	2	0	2	0	0	0	0	1	0	2	1	0	0	0	/	
Novel TSPEAR mutations in non-syndromic oligodontia	2020	Korea	TSPEAR	1	1	2	0	0	0	2	0	0	0	0	0	0	0	2	/	
TSPEAR variants are primarily associated with ectodermal dysplasia and tooth agenesis but not hearing loss: A novel cohort study	2021	Canada,USA,France	TSPEAR	3	4	2	2	4	4	6	0	2	1	2	2	4	6	6	patient#17:dry skin, eczema, taurodontism; patient#18:scoliosis; patient#15: attention disorder, large anteverted ears, prognathism, speech difficulties	ectodermal dysplasia
Aetiological Evaluation of Oligodontia in a Three-Generation Family	2020	Turkey	WNT10A	2	1	0	2	1	0	2	0	3	0	2	1	0	0	2	/	
Novel mutations identified in patients with tooth agenesis by whole - exome sequencing	2019	China	WNT10A	1	2	0	1	2	2	2	0	2	0	0	0	2	2	2	/	
Deleterious Variants in WNT10A, EDAR, and EDA Causing Isolated and Syndromic Tooth Agenesis: A Structural Perspective from MolecularDynamics Simulations	2019	Pakistan, Egypt, Saudi Arabia,	WNT10A	1	2	0	2	2	1	2	0	2	2	1	0	2	2	2	/	
Whole-Exome Sequencing Identifies Novel Variants for Tooth Agenesis	2018	Turkish	WNT10A	5	8	2	6	7	8	9	0	9	3	8	7	7	8	10	/	
Role of WNT10A in failure of tooth development in humans and zebrafish	2017	USA	WNT10A	1	0	2	2	2	1	0	0	0	2	1	0	0	0	1	/	
Dental and extra-oral clinical features in 41 patients with WNT10A gene mutations: A multicentric genotype–phenotype study	2017	French	WNT10A	1	0	0	2	2	0	0	0	0	0	2	2	0	0	0	/	
WNT10A mutations account for ¼ of population-based isolated oligodontia and show phenotypic correlations.	2013	Sweden.	WNT10A	28	10	15	48	30	4	24	0	12	9	42	27	3	6	14	/	
Candidate Gene Analysis of Tooth Agenesis Identifies Novel Mutations in Six Genes and Suggests Significant Role for WNT and EDA Signaling and Allele Combinations	2013	Finland	WNT10A	25	10	0	47	34	15	28	0	13	0	42	20	9	13	16	/	
Eight Mutations of Three Genes (EDA, EDAR, and WNT10A) Identified in Seven Hypohidrotic Ectodermal Dysplasia Patients	2016	China	WNT10A	1	2	0	2	0	2	2	0	1	0	0	0	2	2	2	hypotrichosis,sparse hair,eczema	HED (Hypohidrotic Ectodermal Dysplasia)
Odonto-onycho-dermal dysplasia in a patient homozygous for a WNT10A nonsense mutation and mild manifestations of ectodermal dysplasia in carriers of the mutation	2016	Denmark	WNT10A	1	2	2	2	2	2	2	0	2	2	2	2	2	2	2	onychodysplasia, palmoplantar hyperkeratosis, dry skin, hypotrichosis, and hyperhidrosis of the palms and soles	OODD (odonto-onycho-dermal dysplasia)
Abnormal primary and permanent dentitions with ectodermal symptoms predict WNT10A deficiency	2016	Sweden	WNT10A	7	10	10	12	14	8	14	2	12	7	14	14	7	12	13	hyperhidrosis of palms and soles<br>Coarse hair structure and/or light/scarce/brittle hair<br>Nail abnormalities<br>Dryskin	ectodermal dysplasia
Mutations in WNT10A Are Frequently Involved in Oligodontia Associated With Minor Signs of Ectodermal Dysplasia	2013	France	WNT10A	9	14	4	11	9	7	14	0	12	6	14	10	8	11	12	leukonychia, short stature, hives, flaking, small nose, thin and fragile hair, sparse hair; slow growth,rare eyebrows, no pilosity,hypohidrosis, intolerance to heat, sweating delay, hyperhidrosis,koilonychia, thin nail, periorbital hyperpigmentation, palmoplantar hyperkeratosis, palmoplantar erythema, immune deficiency, infection susceptibility, eczema, dry skin	HED (Hypohidrotic Ectodermal Dysplasia)
WNT10A Mutations Are a Frequent Cause of a Broad Spectrum of Ectodermal Dysplasias with Sex-Biased Manifestation Pattern in Heterozygotes	2009	Lebanese	WNT10A	10	15	8	14	17	17	15	6	14	8	15	10	12	14	17	sparse scalp hair,sparse body hair, sparse eyebrows, Short eyelashes, hypohidrosis,hyperhidrosis, dry skin, soft, thin skin, facial skin erythema, palmoplantar hyperkeratosis, or nail dysplasia	ectodermal dysplasia
Targeted next-generation sequencing (NGS) analysis of mutations in nonsyndromic tooth agenesis candidate genes	2021	Turkey	WNT10A	1	0	2	2	1	2	2	2	0	2	2	0	2	2	2	/	
Mutations in WNT10B Are Identified in Individuals with Oligodontia	2016	China	WNT10B	6	3	3	6	6	7	10	6	3	4	8	1	6	11	8	/	
A novel *PITX2* mutation in non-syndromic orodental anomalies	2017	Thailand	PITX2	1	2	0	0	1	2	2	0	2	0	0	2	2	2	2	/	
Novel Identification of a Four-base-pair Deletion Mutation in PITX2 in a Rieger Syndrome Family	2003	China	PITX2	1	1	2	2	2	2	2	2	0	0	2	2	2	2	2	Rieger Syndrome	
Dental anomalies in Axenfeld–Rieger syndrome	2005	UK	PITX2	1	1	0	0	0	2	2	2	0	0	2	0	0	2	0	Axenfeld–Rieger syndrome	
Novel PITX2 mutations identified in Axenfeld–Rieger syndrome and the pattern of PITX2-related tooth agenesis	2019	China	PITX2	8	11	6	11	9	13	12	16	3	0	9	8	11	11	14	Axenfeld–Rieger syndrome	
Identification of OPN3 as associated with non-syndromic oligodontia in a Japanese population	2021	Janpan	OPN3	1	2	0	1	0	2	2	0	0	0	2	1	2	0	2	/	
BMP4 mutations in tooth agenesis and low bone mass	2019	China	BMP4	3	2	3	4	6	0	4	0	2	2	6	2	1	2	4	/	
Phenotype characterization and sequence analysis of BMP2 and BMP4 variants in two Mexican families with oligodontia	2012	Mexican	BMP4	1	0	0	2	2	0	2	0	2	0	2	0	0	2	0	/	/
Phenotype characterization and sequence analysis of BMP2 and BMP4 variants in two Mexican families with oligodontia	2012	Mexican	BMP2	3	1	1	6	2	0	6	0	2	4	3	0	0	1	6	/	
A novel inhibitor of nuclear factor kappa-B kinase subunit gamma mutation identified in an incontinentia pigmenti patient with syndromic tooth agenesis	2019	China	IKBKG	1	1	0	2	1	0	1	0	1	1	2	2	0	0	0	Reticular hyperpigmentation	incontinentia pigmenti
GREMLIN 2 Mutations and Dental Anomalies	2015	Thailand	GREMLIN 2	1	2	0	2	2	2	2	0	2	0	2	0	2	2	1	/	

List of Articles Analyzed in this Study[1-96]

1 Alfawaz, S. *et al.* Recessive oligodontia linked to a homozygous loss-of-function mutation in the SMOC2 gene. *Arch Oral Biol.* **58**, 462-466, (2013).

2 Arte, S., Parmanen, S., Pirinen, S., Alaluusua, S. & Nieminen, P. Candidate gene analysis of tooth agenesis identifies novel mutations in six genes and suggests significant role for WNT and EDA signaling and allele combinations. *PLoS One* **8**, e73705, (2013).

3 Arzoo, P. S., Klar, J., Bergendal, B., Norderyd, J. & Dahl, N. WNT10A mutations account for (1/4) of population-based isolated oligodontia and show phenotypic correlations. *Am J Med Genet A* **164A**, 353-359, (2014).

4 Beard, C., Purvis, R., Winship, I. M., Macrae, F. A. & Buchanan, D. D. Phenotypic confirmation of oligodontia, colorectal polyposis and cancer in a family carrying an exon 7 nonsense variant in the AXIN2 gene. *Fam Cancer* **18**, 311-315, (2019).

5 Bergendal, B., Klar, J., Stecksén-Blicks, C., Norderyd, J. & Dahl, N. Isolated oligodontia associated with mutations in EDARADD, AXIN2, MSX1, and PAX9 genes. *Am J Med Genet A* **155a**, 1616-1622, (2011).

6 Bergendal, B., Norderyd, J., Zhou, X., Klar, J. & Dahl, N. Abnormal primary and permanent dentitions with ectodermal symptoms predict WNT10A deficiency. *BMC Med Genet* **17**, 88, (2016).

7 Bloch-Zupan, A. *et al.* Homozygosity mapping and candidate prioritization identify mutations, missed by whole-exome sequencing, in SMOC2, causing major dental developmental defects. *Am J Hum Genet.* **89**, 773-781, (2011).

8 Boeira, B. R., Jr. & Echeverrigaray, S. Novel missense mutation in PAX9 gene associated with familial tooth agenesis. *J Oral Pathol Med* **42**, 99-105, (2013).

9 Bohring, A. *et al.* WNT10A mutations are a frequent cause of a broad spectrum of ectodermal dysplasias with sex-biased manifestation pattern in heterozygotes. *Am J Hum Genet* **85**, 97-105, (2009).

10 Bonczek, O. *et al.* Next generation sequencing reveals a novel nonsense mutation in MSX1 gene related to oligodontia. *PLoS One* **13**, e0202989, (2018).

11 Bowles, B. *et al.* TSPEAR variants are primarily associated with ectodermal dysplasia and tooth agenesis but not hearing loss: A novel cohort study. *Am J Med Genet A* **185**, 2417-2433, (2021).

12 Chalabreysse, L. *et al.* A new hypo/oligodontia syndrome: Carvajal/Naxos syndrome secondary to desmoplakin-dominant mutations. *J Dent Res* **90**, 58-64, (2011).

13 Chishti, M. S., Muhammad, D., Haider, M. & Ahmad, W. A novel missense mutation in MSX1 underlies autosomal recessive oligodontia with associated dental anomalies in Pakistani families. *J Hum Genet* **51**, 872-878, (2006).

14 Daw, E. M., Saliba, C., Grech, G. & Camilleri, S. A novel PAX9 mutation causing oligodontia. *Arch Oral Biol* **84**, 100-105, (2017).

15 Dinckan, N. *et al.* Whole-Exome Sequencing Identifies Novel Variants for Tooth Agenesis. *J Dent Res* **97**, 49-59, (2018).

16 Ergün, S. G. *et al.* Aetiological Evaluation of Oligodontia in a Three-Generation Family. *Oral Health Prev Dent* **18**, 271-275, (2020).

17 Fan, Z. *et al.* Novel PITX2 mutations identified in Axenfeld-Rieger syndrome and the pattern of PITX2-related tooth agenesis. *Oral Dis* **25**, 2010-2019, (2019).

18 Frazier-Bowers, S. A., Scott, M. R., Cavender, A., Mensah, J. & D'Souza, R. N. Mutational analysis of families affected with molar oligodontia. *Connect Tissue Res* **43**, 296-300, (2002).

19 Hansen, L., Kreiborg, S., Jarlov, H., Niebuhr, E. & Eiberg, H. A novel nonsense mutation in PAX9 is associated with marked variability in number of missing teeth. *Eur J Oral Sci* **115**, 330-333, (2007).

20 Inagaki, Y. *et al.* Identification of OPN3 as associated with non-syndromic oligodontia in a Japanese population. *J Hum Genet* **66**, 769-775, (2021).

21 Intarak, N. *et al.* A novel PITX2 mutation in non-syndromic oro-dental anomalies. *Oral Dis* **9**, 12804, (2017).

22 Issa, Y. A. *et al.* Mutation of KREMEN1, a modulator of Wnt signaling, is responsible for ectodermal dysplasia including oligodontia in Palestinian families. *Eur J Hum Genet* **24**, 1430-1435, (2016).

23 Jumlongras, D. *et al.* A novel missense mutation in the paired domain of PAX9 causes non-syndromic oligodontia. *Hum Genet* **114**, 242-249, (2004).

24 Kantaputra, P. N. *et al.* GREMLIN 2 Mutations and Dental Anomalies. *J Dent Res* **94**, 1646-1652, (2015).

25 Kapadia, H., Frazier-Bowers, S., Ogawa, T. & D'Souza, R. N. Molecular characterization of a novel PAX9 missense mutation causing posterior tooth agenesis. *Eur J Hum Genet* **14**, 403-409, (2006).

26 Keskin, G., Karaer, K. & Uçar Gündoğar, Z. Targeted next-generation sequencing (NGS) analysis of mutations in nonsyndromic tooth agenesis candidate genes : Analysis of a Turkish cohort. *J Orofac Orthop*, (2021).

27 Khabour, O. F., Mesmar, F. S., Al-Tamimi, F., Al-Batayneh, O. B. & Owais, A. I. Missense mutation of the EDA gene in a Jordanian family with X-linked hypohidrotic ectodermal dysplasia: phenotypic appearance and speech problems. *Genet Mol Res* **9**, 941-948, (2010).

28 Kim, J. W., Simmer, J. P., Lin, B. P. & Hu, J. C. Novel MSX1 Frameshift Causes Autosomal-dominant Oligodontia. *J. Dent. Res.* **85**, 267-271, (2006).

29 Kimura, M. *et al.* Novel nonsense mutation in MSX1 in familial nonsyndromic oligodontia: subcellular localization and role of homeodomain/MH4. *Eur J Oral Sci* **122**, 15-20, (2014).

30 Klein, M. L., Nieminen, P., Lammi, L., Niebuhr, E. & Kreiborg, S. Novel mutation of the initiation codon of PAX9 causes oligodontia. *J Dent Res* **84**, 43-47, (2005).

31 Koskinen, S., Keski-Filppula, R., Alapulli, H., Nieminen, P. & Anttonen, V. Familial oligodontia and regional odontodysplasia associated with a PAX9 initiation codon mutation. *Clin Oral Investig* **23**, 4107-4111, (2019).

32 Krøigård, A. B., Clemmensen, O., Gjørup, H., Hertz, J. M. & Bygum, A. Odonto-onycho-dermal dysplasia in a patient homozygous for a WNT10A nonsense mutation and mild manifestations of ectodermal dysplasia in carriers of the mutation. *BMC Dermatol* **16**, 3, (2016).

33 Lammi, L. *et al.* Mutations in AXIN2 cause familial tooth agenesis and predispose to colorectal cancer. *Am J Hum Genet* **74**, 1043-1050, (2004).

34 Lammi, L. *et al.* A missense mutation in PAX9 in a family with distinct phenotype of oligodontia. *Eur J Hum Genet* **11**, 866-871., (2003).

35 Lee, K. E. *et al.* Oligodontia and curly hair occur with ectodysplasin-a mutations. *J Dent Res* **93**, 371-375, (2014).

36 Liang, J., Qin, C., Yue, H., He, H. & Bian, Z. A novel initiation codon mutation of PAX9 in a family with oligodontia. *Arch Oral Biol* **61**, 144-148, (2016).

37 Liang, J., Song, G., Li, Q. & Bian, Z. Novel missense mutations in PAX9 causing oligodontia. *Arch Oral Biol* **57**, 784-789, (2012).

38 Liedén, A., Kvarnung, M., Nilssson, D., Sahlin, E. & Lundberg, E. S. Intragenic duplication--a novel causative mechanism for SATB2-associated syndrome. *Am J Med Genet A* **164a**, 3083-3087, (2014).

39 Liu, H., Ding, T., Zhan, Y. & Feng, H. A Novel AXIN2 Missense Mutation Is Associated with Non-Syndromic Oligodontia. *PLoS One* **10**, e0138221, (2015).

40 Ma, T. *et al.* A novel mutation of MSX1 inherited from maternal mosaicism causes a severely affected child with nonsyndromic oligodontia. *Ann Hum Genet* **84**, 97-101, (2020).

41 Marvin, M. L. *et al.* AXIN2-associated autosomal dominant ectodermal dysplasia and neoplastic syndrome. *Am J Med Genet A* **155a**, 898-902, (2011).

42 Massink, M. P. *et al.* Loss-of-Function Mutations in the WNT Co-receptor LRP6 Cause Autosomal-Dominant Oligodontia. *Am J Hum Genet* **97**, 621-626, (2015).

43 Mitsui, S. N. *et al.* Novel PAX9 mutations cause non-syndromic tooth agenesis. *J Dent Res* **93**, 245-249, (2014).

44 Mostowska, A., Biedziak, B. & Trzeciak, W. H. A novel c.581C>T transition localized in a highly conserved homeobox sequence of MSX1: is it responsible for oligodontia? *J Appl Genet* **47**, 159-164, (2006).

45 Mostowska, A., Biedziak, B. & Trzeciak, W. H. A novel mutation in PAX9 causes familial form of molar oligodontia. *Eur J Hum Genet* **14**, 173-179, (2006).

46 Mostowska, A., Kobielak, A., Biedziak, B. & Trzeciak, W. H. Novel mutation in the paired box sequence of PAX9 gene in a sporadic form of oligodontia. *Eur J Oral Sci* **111**, 272-276, (2003).

47 Mumtaz, S. *et al.* Novel EDAR mutation in tooth agenesis and variable associated features. *Eur J Med Genet* **63**, 103926, (2020).

48 Mu, Y. *et al.* Phenotype characterization and sequence analysis of BMP2 and BMP4 variants in two Mexican families with oligodontia. *Genet Mol Res* **11**, 4110-4120, (2012).

49 Murakami, A. *et al.* Characterization of PAX9 variant P20L identified in a Japanese family with tooth agenesis. *PLoS One* **12**, e0186260, (2017).

50 Nieminen, P. *et al.* Identification of a nonsense mutation in the PAX9 gene in molar oligodontia. *Eur J Hum Genet* **9**, 743-746, (2001).

51 Nieminen, P. *et al.* MSX1 gene is deleted in Wolf-Hirschhorn syndrome patients with oligodontia. *J Dent Res* **82**, 1013-1017, (2003).

52 Nikopensius, T. *et al.* Non-syndromic tooth agenesis associated with a nonsense mutation in ectodysplasin-A (EDA). *J Dent Res* **92**, 507-511, (2013).

53 O'Dwyer, E. M. & Jones, D. C. Dental anomalies in Axenfeld-Rieger syndrome. *Int J Paediatr Dent* **15**, 459-463, (2005).

54 Parveen, A. *et al.* Deleterious Variants in WNT10A, EDAR, and EDA Causing Isolated and Syndromic Tooth Agenesis: A Structural Perspective from Molecular Dynamics Simulations. *Int J Mol Sci* **20**, (2019).

55 Pawlowska, E., Janik-Papis, K., Poplawski, T., Blasiak, J. & Szczepanska, J. Mutations in the PAX9 gene in sporadic oligodontia. *Orthod Craniofac Res* **13**, 142-152, (2010).

56 Pawlowska, E., Janik-Papis, K., Wisniewska-Jarosinska, M., Szczepanska, J. & Blasiak, J. Mutations in the human homeobox MSX1 gene in the congenital lack of permanent teeth. *Tohoku J Exp Med* **217**, 307-312, (2009).

57 Plaisancié, J. *et al.* Mutations in WNT10A are frequently involved in oligodontia associated with minor signs of ectodermal dysplasia. *Am J Med Genet A* **161a**, 671-678, (2013).

58 Qin, H. & Cai, J. Axis inhibition protein 2 polymorphisms may be a risk factor for families with isolated oligodontia. *Mol Med Rep* **11**, 1899-1904, (2015).

59 Qin, H., Xu, H. Z. & Xuan, K. Clinical and genetic evaluation of a Chinese family with isolated oligodontia. *Arch Oral Biol* **58**, 1180-1186, (2013).

60 Ross, J. *et al.* Concurrent manifestation of oligodontia and thrombocytopenia caused by a contiguous gene deletion in 12p13.2: A three-generation clinical report. *Mol Genet Genomic Med* **7**, e679, (2019).

61 Ruiz-Heiland, G. *et al.* Novel missense mutation in the EDA gene in a family affected by oligodontia. *J Orofac Orthop* **77**, 31-38, (2016).

62 Sarkar, T., Bansal, R. & Das, P. Whole genome sequencing reveals novel non-synonymous mutation in ectodysplasin A (EDA) associated with non-syndromic X-linked dominant congenital tooth agenesis. *PLoS One* **9**, e106811, (2014).

63 Sarkar, T., Bansal, R. & Das, P. A novel G to A transition at initiation codon and exon-intron boundary of PAX9 identified in association with familial isolated oligodontia. *Gene* **635**, 69-76, (2017).

64 Šerý, O. *et al.* A screen of a large Czech cohort of oligodontia patients implicates a novel mutation in the PAX9 gene. *Eur J Oral Sci* **123**, 65-71, (2015).

65 Shahid, M. *et al.* Mutations in MSX1, PAX9 and MMP20 genes in Saudi Arabian patients with tooth agenesis. *Eur J Med Genet* **59**, 377-385, (2016).

66 Song, S. *et al.* EDA gene mutations underlie non-syndromic oligodontia. *J Dent Res* **88**, 126-131, (2009).

67 Song, J. S., Bae, M. & Kim, J. W. Novel TSPEAR mutations in non-syndromic oligodontia. *Oral Dis* **26**, 847-849, (2020).

68 Sun, S. *et al.* A novel inhibitor of nuclear factor kappa-B kinase subunit gamma mutation identified in an incontinentia pigmenti patient with syndromic tooth agenesis. *Arch Oral Biol* **101**, 100-107, (2019).

69 Sun, K. *et al.* Functional study of novel PAX9 variants: The paired domain and non-syndromic oligodontia. *Oral Dis*, (2020).

70 Tallón-Walton, V. *et al.* Identification of a novel mutation in the PAX9 gene in a family affected by oligodontia and other dental anomalies. *Eur J Oral Sci* **115**, 427-432, (2007).

71 Tardieu, C. *et al.* Dental and extra-oral clinical features in 41 patients with WNT10A gene mutations: A multicentric genotype-phenotype study. *Clin Genet* **92**, 477-486, (2017).

72 Tatematsu, T. *et al.* An aberrant splice acceptor site due to a novel intronic nucleotide substitution in MSX1 gene is the cause of congenital tooth agenesis in a Japanese family. *PLoS One* **10**, e0128227, (2015).

73 Thimmegowda, U., Prasanna, P., Athimuthu, A., Bhat, P. K. & Puttashamachari, Y. A Nonsyndromic Autosomal Dominant Oligodontia with A Novel Mutation of PAX9-A Clinical and Genetic Report. *J Clin Diagn Res* **9**, Zd08-10, (2015).

74 Wang, Y., Zhao, H., Zhang, X. & Feng, H. Novel identification of a four-base-pair deletion mutation in PITX2 in a Rieger syndrome family. *J Dent Res* **82**, 1008-1012, (2003).

75 Wang, J. *et al.* Sequence analysis of PAX9, MSX1 and AXIN2 genes in a Chinese oligodontia family. *Arch Oral Biol* **56**, 1027-1034, (2011).

76 Wang, S. K., Chan, H. C., Makovey, I., Simmer, J. P. & Hu, J. C. Novel PAX9 and COL1A2 missense mutations causing tooth agenesis and OI/DGI without skeletal abnormalities. *PLoS One* **7**, e51533, (2012).

77 Wang, Y. *et al.* Identification and functional analysis of two novel PAX9 mutations. *Cells Tissues Organs* **189**, 80-87, (2009).

78 Wang, H. *et al.* A novel missense mutation of LRP6 identified by whole-exome sequencing in a Chinese family with non-syndromic tooth agenesis. *Orthod Craniofac Res* **24**, 233-240, (2021).

79 Wong, S. W. *et al.* Nine Novel PAX9 Mutations and a Distinct Tooth Agenesis Genotype-Phenotype. *J Dent Res* **97**, 155-162, (2018).

80 Wong, S. *et al.* Novel missense mutations in the AXIN2 gene associated with non-syndromic oligodontia. *Arch Oral Biol* **59**, 349-353, (2014).

81 Wong, S. W. *et al.* A novel non-stop mutation in MSX1 causing autosomal dominant non-syndromic oligodontia. *Mutagenesis* **29**, 319-323, (2014).

82 Xin, T. *et al.* A novel mutation of MSX1 in oligodontia inhibits odontogenesis of dental pulp stem cells via the ERK pathway. *Stem Cell Res Ther* **9**, 221, (2018).

83 Xuan, K. *et al.* Identification of a novel missense mutation of MSX1 gene in Chinese family with autosomal-dominant oligodontia. *Arch Oral Biol* **53**, 773-779, (2008).

84 Xue, J. *et al.* A novel MSX1 intronic mutation associated with autosomal dominant non-syndromic oligodontia in a large Chinese family pedigree. *Clin Chim Acta* **461**, 135-140, (2016).

85 Yamaguchi, S. *et al.* Characterization of novel MSX1 mutations identified in Japanese patients with nonsyndromic tooth agenesis. *PLoS One* **9**, e102944, (2014).

86 Yang, L., Liang, J., Yue, H. & Bian, Z. Two novel mutations in MSX1 causing oligodontia. *PLoS One* **15**, e0227287, (2020).

87 Yu, P. *et al.* Mutations in WNT10B Are Identified in Individuals with Oligodontia. *Am J Hum Genet* **99**, 195-201, (2016).

88 Yu, M. *et al.* BMP4 mutations in tooth agenesis and low bone mass. *Arch Oral Biol* **103**, 40-46, (2019).

89 Yu, M. *et al.* Lrp6 Dynamic Expression in Tooth Development and Mutations in Oligodontia. *J Dent Res* **100**, 415-422, (2021).

90 Yuan, Q. *et al.* Role of WNT10A in failure of tooth development in humans and zebrafish. *Mol Genet Genomic Med* **5**, 730-741, (2017).

91 Yue, H., Liang, J., Yang, K., Hua, B. & Bian, Z. Functional analysis of a novel missense mutation in AXIN2 associated with non-syndromic tooth agenesis. *Eur J Oral Sci* **124**, 228-233, (2016).

92 Zeng, B. *et al.* Eight Mutations of Three Genes (EDA, EDAR, and WNT10A) Identified in Seven Hypohidrotic Ectodermal Dysplasia Patients. *Genes (Basel)* **7**, (2016).

93 Zhao, K. *et al.* Novel mutations identified in patients with tooth agenesis by whole-exome sequencing. *Oral Dis* **25**, 523-534, (2019).

94 Zhang, L. *et al.* Comparative analysis of rare EDAR mutations and tooth agenesis pattern in EDAR- and EDA-associated nonsyndromic oligodontia. *Hum Mutat* **41**, 1957-1966, (2020).

95 Zhang, H. *et al.* A novel EDAR missense mutation identified by whole-exome sequencing with non-syndromic tooth agenesis in a Chinese family. *Mol Genet Genomic Med* **9**, e1684, (2021).

96 Zheng, J. *et al.* Novel MSX1 variants identified in families with nonsyndromic oligodontia. *Int J Oral Sci* **13**, 2, (2021).
